# Structure of the heterotrimeric membrane protein complex FtsB-FtsL-FtsQ of the bacterial divisome

**DOI:** 10.1038/s41467-023-37543-4

**Published:** 2023-04-05

**Authors:** Hong Thuy Vy Nguyen, Xiaorui Chen, Claudia Parada, An-Chi Luo, Orion Shih, U-Ser Jeng, Chia-Ying Huang, Yu-Ling Shih, Che Ma

**Affiliations:** 1 Genomics Research Center, Academia Sinica, Taipei, 115 Taiwan; 2 Chemical Biology and Molecular Biophysics program, Taiwan International Graduate Program, Academia Sinica, Taipei, 115 Taiwan; 3 Institute of Biochemical Sciences, National Taiwan University, Taipei, 10617 Taiwan; 4 Institute of Biological Chemistry, Academia Sinica, Taipei, 115 Taiwan; 5 National Synchrotron Radiation Research Center, Hsinchu, 30076 Taiwan; 6 Department of Chemical Engineering, National Tsing Hua University, Hsinchu, 30044 Taiwan; 7 Paul Scherrer Institute, Forschungsstrasse 111, Villigen-PSI, 5232 Switzerland

**Keywords:** X-ray crystallography, Bacteria, Proteins, Membrane structure and assembly

## Abstract

The synthesis of the cell-wall peptidoglycan during bacterial cell division is mediated by a multiprotein machine, called the divisome. The essential membrane protein complex of FtsB, FtsL and FtsQ (FtsBLQ) is at the heart of the divisome assembly cascade in *Escherichia coli*. This complex regulates the transglycosylation and transpeptidation activities of the FtsW-FtsI complex and PBP1b via coordination with FtsN, the trigger for the onset of constriction. Yet the underlying mechanism of FtsBLQ-mediated regulation is largely unknown. Here, we report the full-length structure of the heterotrimeric FtsBLQ complex, which reveals a V-shaped architecture in a tilted orientation. Such a conformation could be strengthened by the transmembrane and the coiled-coil domains of the FtsBL heterodimer, as well as an extended β-sheet of the C-terminal interaction site involving all three proteins. This trimeric structure may also facilitate interactions with other divisome proteins in an allosteric manner. These results lead us to propose a structure-based model that delineates the mechanism of the regulation of peptidoglycan synthases by the FtsBLQ complex.

## Abstract (web summary)

The FtsB-FtsL-FtsQ complex is essential for regulating cell wall synthesis during bacterial cell division. Here, authors report the full-length trimeric structure showing a tilted V-shaped conformation and suggest an allosteric regulatory mechanism.

## Introduction

**Fig. 1:**
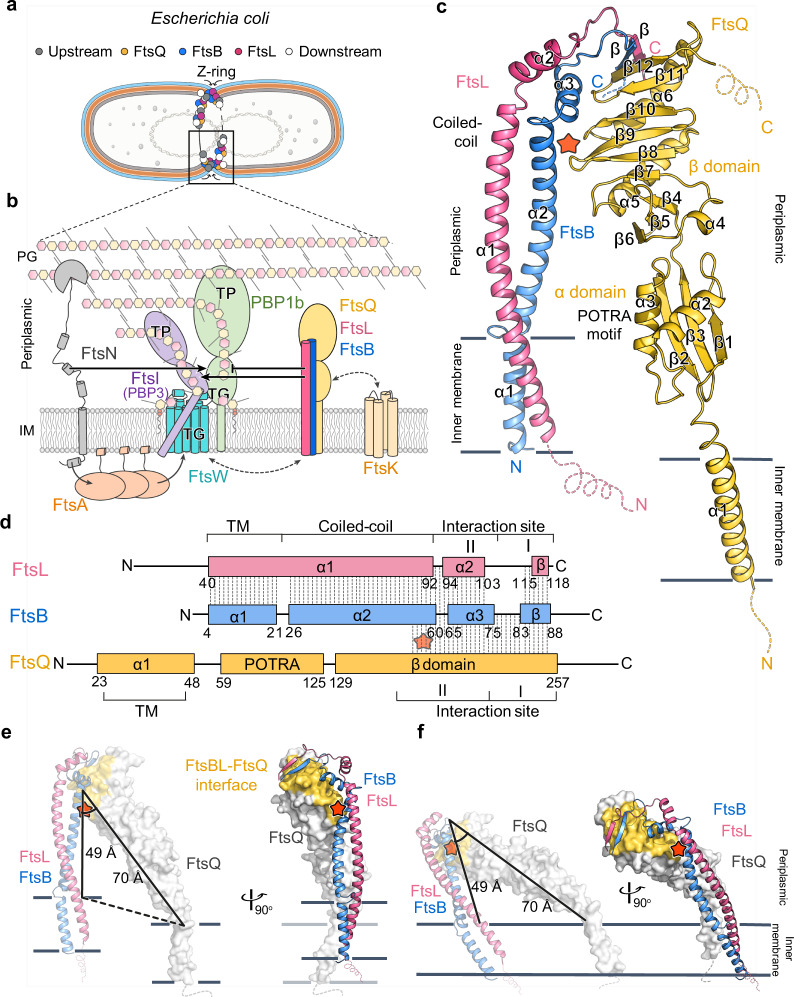
The FtsB-FtsL-FtsQ (FtsBLQ) complex in the bacterial divisome and its overall structure. **a** A schematic view of the recruitment of FtsBLQ to the divisome in *E. coli*. Divisome proteins are shown as circles: FtsB (blue); FtsL (pink); FtsQ (yellow); the upstream (black) and downstream (white) proteins of FtsBLQ. The inner and outer membranes and the periplasm are shown in grey, light blue, and orange layers. **b** A cartoon model of the molecular relationship between key divisome proteins at the division site: FtsBLQ (blue, pink, and yellow), FtsK (only TM domain shown, wheat), PBP1b (green), FtsW-FtsI (cyan and purple), FtsN (grey) and FtsA (orange). Solid black arrows/lines indicate the activating/inhibitory regulations. Dashed lines with arrows indicate the recruitment. The inner membrane (IM) is drawn as a grey lipid bilayer, and peptidoglycan (PG) sugars as hexagons. **c** Overall structure of the FtsBLQ complex as an inverted “V” shape, showing FtsB (blue), FtsL (pink), and FtsQ (yellow) in a stoichiometry of 1:1:1, with each domain accordingly labeled. All secondary structural elements are numbered sequentially. Missing stretches of residues, including residues 90–103 of FtsB; 1–39 of FtsL; 1–21, and 259–276 of FtsQ, are drawn as dashed lines. **d** Domain organization of FtsB, FtsL, and FtsQ with dash lines indicating the observed interactions between each other within the complex. The TM, the coiled coil, the interaction sites, the N- and C- termini, and the start and end of each structure element are also labeled. **e** The length difference between the periplasmic parts of FtsBL and FtsQ is shown in the same view as (**c**) - left or rotated for 90° - right. **f** The tilted FtsBLQ structure that fits all TM domains in the membrane, in the same views as in (**e**). In **c**–**f** the new FtsB-FtsQ interface is highlighted with an orange star.

Bacterial cell division is a fundamental process which harbors a rich source of potential antibiotic targets^1,2^. In the current era, with the increasing prevalence of antibiotic resistance in bacteria, antibiotics with novel mechanisms are urgently called for. Exploitation of the cell division process is hence an intriguing direction but nonetheless limited by the lack of structural information of the multi-protein division machinery, called the divisome^1,2^. In the case of *Escherichia coli* (*E. coli*), this complex is recruited to the division site at the mid-cell (Fig. 1a)^3–5^. A core complex at the heart of this machinery is composed of three type-II bitopic membrane proteins, the Filamentous temperature-sensitive proteins B, L and Q (FtsB, FtsL, FtsQ) (Fig. 1b). This complex is recruited by the chromosome segregator FtsK, and is responsible for recruiting the peptidoglycan (PG) synthase FtsW-FtsI (FtsWI) complex, which contains the transglycosylase (TG) FtsW and the transpeptidase (TP) FtsI (alternatively PBP3, Penicillin Binding Protein 3)^6,7^. FtsN is then recruited upon the assembly of all these proteins, which activates the septal PG synthesis and triggers the onset of cell constriction^8,9^.

For a long time, the FtsBLQ complex was believed to act as a non-enzymatic scaffold for the assembly of the divisome, but recent studies revealed it to be a positive regulator of the FtsWI complex and a negative regulator of the bi-functional PG synthase PBP1b (Fig. 1b)^10–12^. The periplasmic interaction between FtsI and FtsL was reported as the key for FtsWI activation^11^, and the suppression of PBP1b activity by FtsL was proposed to be competed with and released by FtsN^13^. However, it still remains elusive how the multirole FtsBLQ complex fulfills its regulatory function at the molecular level, and how it influences other divisome proteins through direct and indirect interactions, such as FtsA^9^, FtsN^14^, etc. Moreover, while the PG synthases have been extensively studied as antibiotic targets for decades, their regulators (FtsB, L, and Q) are much less explored, although they are also proposed as potential targets^3,15–17^. The lack of a complete atomic view of the FtsBLQ complex has become one of the major hinderances to a further mechanistic understanding, and to an inspiration for the antimicrobial drug design from the structural insights of divisome proteins.

In this work, we report the structure of the full-length heterotrimeric membrane protein complex of FtsB-FtsL-FtsQ (the FtsBLQ complex). The structure resembles an inverted “V” with two branches of different lengths. The two branches form a rigid acute angle with each other, which is strengthened by an interface between FtsB and FtsQ. Such a conformation implicates a likely tilted orientation when the complex is inserted in the membrane, and mutating key functional residues at the interaction sites resulted in differential impacts on the complex formation and cell division. A potential allosteric change that disengages the FtsB-FtsQ interface is proposed as a structural model to regulate the septal PG synthesis via modulating the interaction between the FtsBLQ complex and other division-related proteins.

## Results

### Overall structure of the FtsB-FtsL-FtsQ complex

The crystal structure of the FtsBLQ complex was determined at 3.1 Å resolution by molecular replacement using the partial structure of the complex as the input model, which contains the periplasmic domain of FtsQ and the C-terminal region of FtsB (PDB: 5Z2W^18^) (Supplementary Table 1). The remaining atoms (~40%) were manually built, including three fully resolved transmembrane (TM) helices. The three proteins of the complex, in a stoichiometry of 1:1:1, meet at the C-terminal membrane-distal region and form an inverted V-shape conformation (Fig. 1c, Supplementary Fig. 1). One branch of the V-shape is a lengthy coiled-coil heterodimer FtsB-FtsL (FtsBL), which extends continuously from the inner membrane to the periplasmic, where their key functional domains locate (Fig. 1c). The other branch comes from FtsQ that adopts a rod-like shape, with two globular periplasmic domains arranged linearly and connected to the TM helix α1 in a slightly bent manner (Supplementary Fig. 2a–c). Although the previous two-hybrid assay and the photo cross-linking data proposed additional interaction hotspots between FtsBL and FtsQ^19,20^, these residues are solvent exposed in the current structure, and their double or triple mutants (FtsQ^S166R-E141V^, FtsQ^K59E-R75E-Q76A^) do not disrupt the complex formation in vitro (Supplementary Fig. 2d).

A notable feature of the V-shaped FtsBLQ structure is that the length of each branch is different: the FtsBL branch spans ~100 Å and the FtsQ branch ~120 Å (Supplementary Fig. 1). The crossing point of the two branches, however, is not at the very tip but somewhere in the middle of the continuous FtsBL coiled coil (Fig. 1d), forming an included angle of ~37°, where an interface between FtsB and FtsQ is revealed (Fig. 1c-e, Supplementary Fig. 1). In the other branch, the two globular domains of FtsQ, the Polypeptide Transport-Associated (POTRA) α domain and the C-terminal β domain, are rigidly connected together with the lowest B-factors observed at their interface (Supplementary Fig. 2b), and the least structural variation compared with the previous structures of the periplasmic part of FtsQ (PDB: 2VH1^21^, 6H9N^22^ and 5Z2W^18^) (Supplementary Fig. 2a). Residues with the highest B-factors are found at the juxta-membrane linkers (α2-α3, β2-β3 and TM-β1) which are loosely packed (Supplementary Fig. 2c), suggesting that a swing motion is possible for the whole periplasmic part of FtsQ relative to its membrane anchor. Therefore, the two rigid periplasmic branches of FtsBLQ form an obtuse triangle with the plane of the membrane (49 Å and 70 Å in length and at ~37° angle) and are connected to the bendable FtsQ-TM and the unbendable FtsBL-TM on each side (Fig. 1e). Such a structure would most likely tilt down when inserted in the membrane (Fig. 1f), and a structural rearrangement to achieve a perpendicular insertion is possible yet at a cost of disrupting the protein-protein interface (Supplementary Fig. 2e).

### The transmembrane domains of the FtsB-FtsL-FtsQ complex

**Fig. 2:**
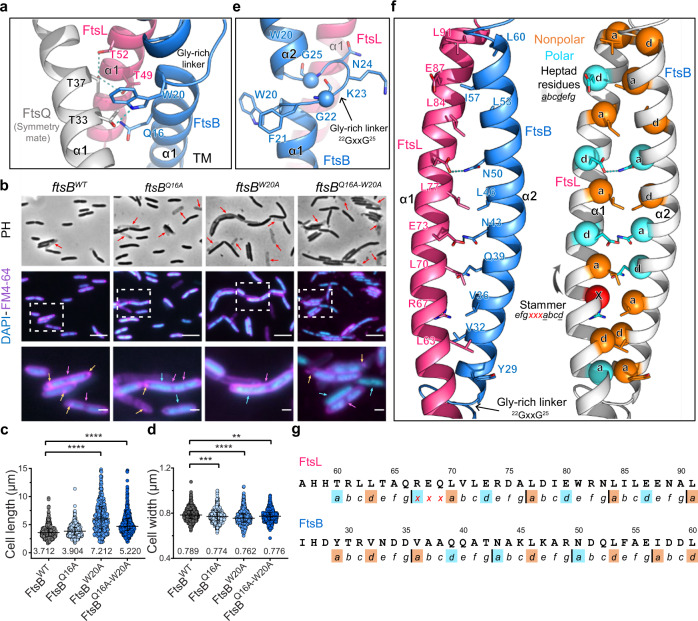
Structural and functional studies of the transmembrane and the coiled-coil domains of the FtsBLQ heterotrimer. **a** Potential interacting residues between FtsB (blue), FtsL (pink) with FtsQ (grey) from its symmetry mate at the transmembrane regions. Residues are shown in sticks and labeled in according colors. **b** Phenotypes of the *ftsB*-knock-down strain by CRISPRi and its complementation by *ftsB*^WT^, *ftsB*^Q16A^, *ftsB*^W20A^ or *ftsB*^Q16A-W20A^ mutants. PH: Phase contrast; Blue DAPI: chromosome stain; Purple FM4-64: membrane stain. Arrows indicate the lysed cells (red), unsegregated chromosome (cyan), lack of septum (magenta), and membrane patches (orange). Scale bar, 5 μm (middle column) and 1 μm for enlarged views of the white box. **c**, **d** The cell length (**c**) and cell width (**d**) of the *ftsB*^*WT*^- or mutant-complemented cells as in (**b**). The comparison was performed by One-way ANOVA test. Data are presented as median (shown on the bottom) with an interquartile range. FtsB^WT^: *n* = 564; FtsB^Q16A^: *n* = 521, *P* = 0.0001 (width); FtsB^W20A^: *n* = 543, *P* < 0.0001 (length, width); FtsB^Q16A-W20A^: *n* = 522, *P* < 0.0001 (length), *P* = 0.0011 (width). **e** The ^22^GxxG^25^ linker region at the connection between the TM and periplasmic parts of FtsB. Residues are drawn as sticks and labeled. **f** Interacting residue pairs of the heterodimeric FtsBL coiled coil. The Gly-rich linker (GxxG) of FtsB is also labeled. The stammer insertion (red) is shown in FtsL, and the core *a* and *d* positions of each heptad are shown as Cα spheres. Polar (cyan) and nonpolar (orange) residues are in sticks and colored accordingly. **g** The primary sequence of FtsB and FtsL in the coiled-coil region with *a* and *d* heptad residues and the stammer insertion colored as in (**f**).

The crystal packing of the FtsBLQ complex reveals that the TM domains of FtsB-FtsL in one asymmetric unit may interact with the TM domain of FtsQ in the neighboring asymmetric unit (Fig. 2a). It results in a buried area of 446.21 Å² with strong electron densities, with the dimerization of two tilted trimers and all TM domains fitting in the same plane of the membrane (Supplementary Fig. 3a–c). Such a tilted conformation agrees with the above-mentioned arrangement, as well as the genetic evidence that the recruiting residues of FtsQ, including V92, Q108, V111, K113, are speculated to face towards the periplasmic loops of FtsK, the upstream DNA translocase in the divisome assembly cascade^21,23,24^ (Supplementary Fig. 3d, e). It also raises the question of whether the crystal packing hints at a possible heterohexameric FtsBLQ in the 2:2:2 stoichiometry^18,25^. As far as being tested in this study, the purified full-length complex exhibited two bands at ~120 kDa and ~240 kDa on a native PAGE (polyacrylamide gel electrophoresis) (Supplementary Fig. 4a), and its molecular weight was determined to be 188 kDa by multi-angle light scattering coupled with size exclusion chromatography (SEC-MALS), which was close to the summed size of a hexamer (117 kDa including the hexa-histidine-tag) and the micelle of 5-Cyclohexylpentyl β-D-maltoside (~23 kDa) (Supplementary Fig. 4b). The SAXS envelop of the full-length complex was found to be big enough to contain two trimers (Supplementary Fig. 4c), and the hexamer also fit better into the experimental SAXS curve than the trimer (Supplementary Fig. 4d). Although no conclusion could yet be made for the oligomeric state of the FtsBLQ complex, it appears likely to be higher than a heterotrimer in vitro, and whether this tendency of oligomerization may play a role in vivo awaits further explorations.

Two other interesting features are observed in the TM domains of FtsB, FtsL, and FtsQ. First, their single-pass TM helices all carry a group of polar residues, most of which are conserved (Supplementary Fig. 5). At the crystal packing interface, the TM helices of FtsBL in one symmetry mate and FtsQ in another present the polar residues that together form a hydrophilic core near the TM midpoint, involving FtsQ^T33, T37^, FtsL^T49, T52^, and FtsB^Q16^, and FtsB^W20^ that interacts with FtsQ^T33^ (Fig. 2a). Even though this hydrophilic core might represent a crystal packing artefact, these residues may still be involved in interactions with other proteins in vivo, lest their polar side chains are unfavorably exposed in the lipid bilayer^26^. Second, the TM domains of FtsB and FtsL are very short, containing only 18 amino acids each (Fig. 1d, Supplementary Fig. 5). Although it is known that a short TM helix tends to adopt a perpendicular membrane insertion^27^, it is less likely for FtsBL due to the overall tilted orientation of the FtsBLQ complex (Fig. 1f). This may in turn lead to a tension between the biological environment and the structural constraint, which could possibly trigger a conformational change when external forces are present^28^.

Mutagenesis studies were performed to understand the contribution of these polar residues to the biochemical properties of the FtsBLQ complex. Mutations such as FtsQ^T37W^ and FtsB^Q16A-W20A^ did not disassemble the complex during co-purification (Supplementary Fig. 4a), but the FtsBLQ^T37W^ mutant complex formed even higher-order oligomers in native PAGE and SEC-MALS (Supplementary Fig. 4a, f), while FtsB^Q16A-W20A^LQ exhibited a broader peak on SEC (Supplementary Fig. 4e). The results suggest that the mutation FtsQ^T37W^ could induce more inter-oligomer interactions and the mutation FtsB^Q16A-W20A^ could affect the homogeneity of the oligomeric states of the complex in vitro. These changes may come from an increased hydrophobicity in the TM domains, or from a different way that the TM helices are inserted in the membrane due to mutations.

To further examine the in vivo effect of mutating these polar residues, a CRISPRi system was employed for phenotypic studies by knocking down (KD) the essential gene, *ftsB*, from the chromosome (Supplementary Fig. 6a–c). The depletion of *ftsB* not only showed growth defects, but was also severely defective in cell division, showing elongated and filamentous phenotypes (Supplementary Fig. 6d, e). The division defects and the transcription level were largely rescued by expressing the wild-type *ftsB* from a complementing plasmid (Supplementary Fig. 7a–d). The *ftsB*-depleted cell was accompanied by abnormally segregated chromosomes and unevenly stained membrane patches of the lipophilic dye FM4-64 (Supplementary Fig. 7d). Despite the unexpected polar effect on the downstream *ispD* and *ispF* genes that could have affected the cell survival (Supplementary Fig. 6c), the ability to rescue cell division in the knock-down strain was sufficient to examine the function of the mutant *ftsB* in cell division (Supplementary Fig. 7d, e). While the *ftsB*^Q16A^ mutant did not cause significant division defects in the complementation experiment, the *ftsB*^W20A^ and *ftsB*^Q16A-W20A^ mutants displayed significantly elongated cells with abnormal chromosome segregation (Fig. 2b–d, Supplementary Fig. 7d, e). These results suggest that the FtsB^W20A^ mutation could affect cell division in vivo by disrupting the interaction either with FtsQ as in symmetry contacts or with other divisome proteins, which was not realized before probably due to different experimental settings^29^.

### The heterodimeric coiled-coil domain of FtsB-FtsL

Continuously extending from the FtsB-FtsL TM domain is a parallel heterodimeric coiled coil, containing ~30 amino acids in each coil (Fig. 2f). The boundary of the coiled-coil segment is defined by a Gly-rich linker (^22^GxxG^25^ ) of FtsB at the N-terminus^29,30^, and two flexible loops at the C-terminus, the α2-α3 linker of FtsB and the α1-α2 linker of FtsL (Fig. 1d, Supplementary Fig. 8a, b). The GxxG linker induces a kink at the connection between the TM and periplasmic parts of FtsB, with two hydrophilic residues (K23 and N24) in the middle and two aromatic residues (W20 and F21) preceding it (Fig. 2e). This kink enables FtsB to wind around the continuous helix of FtsL-α1, and possibly serves as a buoy for the rod-shaped coiled coil to be properly embedded in the membrane (Fig. 1c). The C-terminal end of the coiled coil is linked by 3-to-6-aa loops to two short helices, FtsB-α3 and FtsL-α2, which were previously predicted as the slightly bent “extended coils”, part of the entire FtsBL coiled coil^29,31^. However, in the crystal structure, the FtsB-α3 helix forms a tight bend (Supplementary Fig. 8a), which is not a result of crystal packing (Supplementary Fig. 3a) but is involved in the extensive FtsB-FtsQ interaction (Fig. 1d) and will be described later.

The coiled-coil domain can be grouped into five (for FtsB) or four (for FtsL) heptad repeats, (*abcdefg*)_n_, except for a three-residue stammer inserted between the first and second heptads of FtsL (Fig. 2f, g). The presence of this stammer was not recognized before in the coiled-coil prediction based on the primary sequence^9,29^, but is now revealed in the structural model by a detailed examination of each buried *a* and *d* interacting pair (Fig. 2g). The presence of the stammer helps to resolve the previous controversy about heptad prediction^9,29^, and is proposed to be responsible for the over-winding conformation of the FtsBL coiled coil, introducing more structural flexibility into the coiled coil^32^ (Supplementary Fig. 8c).

Unlike most coiled coils that are stabilized by hydrophobic packing of the core *a* and *d* residues, FtsBL contains alternating hydrophobic and hydrophilic *a-d* interaction pairs (Fig. 2f, Supplementary Table 2). Mutating the hydrophilic residues to hydrophobic ones was found to improve the thermal stability of FtsBL but disrupt the cell division^31^. These buried polar residues, the stammer insertion, and the loose loops at both ends of the coiled-coil segment could enable a short-range sliding of FtsB along the continuous FtsL-α1 when the intra-coiled-coil contacts are transiently dislodged, a case inspired by other similar coiled-coil structures^32,33^. These structural features also implicate the intrinsic flexibility of the coiled coil that could be biologically important for the FtsBLQ complex, pointing towards the dynamic nature of the regulatory function as suggested by several recent studies^9,14,31^. Such a dynamic regulation offers mechanistic insights that a potential movement could occur, which in turn alters the surface accessibility of key functional residues that are mainly located near the C-terminus of the coiled coil (Fig. 1d).

### The membrane-distal interaction site of the FtsB-FtsL-FtsQ complex

**Fig. 3:**
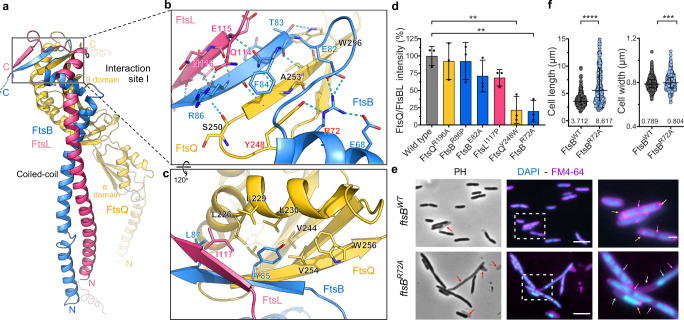
The C-terminal interaction site I of FtsB, FtsL, and FtsQ forms an extended β-sheet. **a** Overall structure of the FtsBLQ complex highlighting the C-terminal interaction site I in a box. FtsB: blue, FtsL: pink, FtsQ: yellow. **b** An enlarged view of the detailed interface as in the box in (**a**), showing the interacting residues in sticks and hydrogen bonds in blue dashed lines. Residues that affect complex formation are labeled in red. **c** A rotated view (120°) of (**b**) showing primarily hydrophobic interactions. **d** The ratio of FtsQ over FtsBL in the three-protein co-purification with different mutations as listed (Supplementary Fig. 2d). The ratio of FtsQ over FtsBL in the wild-type complex is defined as 100%. Results are the mean intensity values of SDS-PAGE bands from three biological replicates. The comparison was performed by One-way ANOVA test. Data are presented as mean with SD. *P* = 0.0054 (FtsQ^Y248W^), *P* = 0.0044 (FtsB^R72A^). **e** Complementation of the *ftsB*-knock-down strain by *ftsB*^*R72A*^ mutant, showing the elongated cell shape. PH: Phase contrast; Blue DAPI: chromosome stain; Purple FM4-64: membrane stain. Arrows indicate the unsegregated chromosome (cyan), void of chromosome (white), lack of septum (magenta), and membrane punctate (orange) and the lysed cell (red). Blue: chromosome stain; Purple: membrane stain. Scale bar, 5 μm (middle column), and 1 μm for enlarged views of the white boxes. **f** The cell length (left) and cell width (right) of the *ftsB*^*WT*^- or mutant-complemented cells as in (**e**). Comparison was performed by two-tailed *t*-test. Data are presented as median (shown on bottom) with interquartile range. Sample size *n* = 564 (FtsB^WT^), *n* = 552 (FtsB^R72A^). *P* < 0.0001 (length), *P* = 0.0001 (width).

FtsB, FtsL, and FtsQ form an extensive interaction site at the membrane-distal region, which can be further divided into Site I and Site II (Fig. 1d). In Site I, a heterotrimeric β-sheet is revealed for the first time in the FtsBLQ complex, involving three C-terminal β-strands, FtsL^E115–V118^, FtsB^E83–P88^, FtsQ^S250–W256^ (Fig. 3a). The previously known FtsB-FtsQ interface in this region^18,22^ is further stabilized by the addition of the FtsL β-strand. On one side of this β-sheet, extensive polar contacts are observed, including FtsL^Q114^, FtsL^N116^, FtsB^R86^, FtsB^R72^, and FtsQ^Y248^ (Fig. 3b), while nonpolar contacts are found on the other side, represented by FtsL^I117^, FtsB^Y85^, FtsQ^W256^, etc. FtsQ^Y248^ further merges with the inner hydrophobic core of the β-domain of FtsQ (Fig. 3c). FtsQ^Y248^ also forms a T-shaped π–π stacking with FtsB^F84^ and interacts with FtsB^R72^ via a hydrogen bond (Fig. 3b). Mutagenesis studies were performed to elucidate key residues required for the complex formation. Single mutants FtsB^R72A^ and FtsQ^Y248W^ were found to cause a significant loss of FtsQ association (80 and 79%, respectively) during co-purification (Fig. 3d, Supplementary Fig. 2d). Other mutants such as FtsB^E82A^, FtsB^R86P^, and FtsL^I117P^ exhibited only minor effects, for their locations may not be critical enough to disengage the assembly of FtsQ onto FtsBL (Fig. 3d, Supplementary Fig. 2d, Supplementary Table 2). The biological importance of FtsB^R72^ is consistent with the previous finding^22^ and reinforced in our in vivo knock-down assay, as its substitution with alanine resulted in significantly longer cells in comparison with the *ftsB*^WT^-complemented cells (Fig. 3e, f, Supplementary Fig. 7d). For some unknown reason, the transcription level of *ftsB*^R72A^ remained low in the complementation experiments even with stronger induction (Supplementary Fig. 7e). In all, key residues in Site I, particularly FtsB^R72^ and FtsQ^Y248^, are crucial in holding the three proteins tightly together^21,34,35^.

**Fig. 4:**
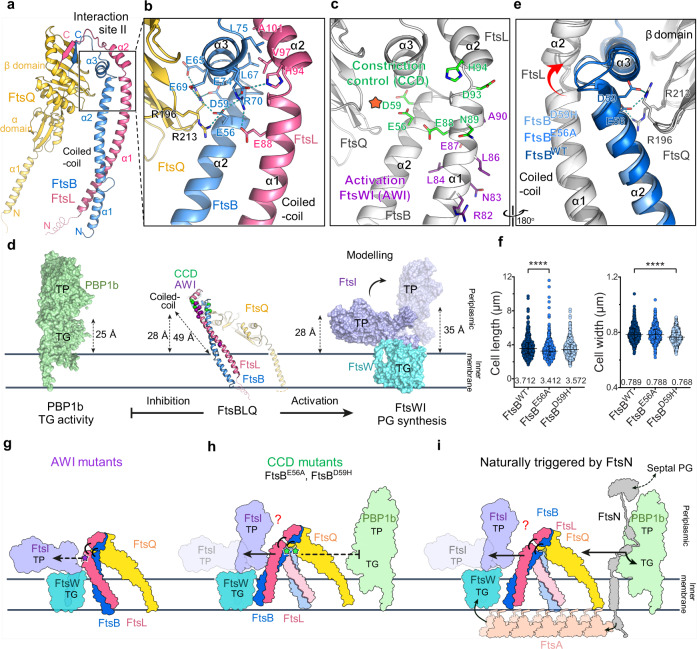
Key functional residues in the interaction site II and a hypothetic model for the regulation of PG synthases. **a** Overall structure of the FtsBLQ complex highlighting the interaction site II in a box. **b** An enlarged view of the box in (**a**), labeling two short helices of FtsB (α2) and FtsL (α3) and the coiled coil, with interacting residues shown in sticks and hydrogen bonds in blue dashed lines. **c** Key residues of the Constriction Control Domain (CCD, green) and the Activation of FtsW-FtsI Domain (AWI, purple) overlap with the interaction site II. FtsB-FtsQ interface: orange star. **d** Distances measured from the membrane plane to the position of CCD and AWI residues of the FtsBLQ complex (middle, ribbon), to the upper side of the TG domain of PBP1b (5HLB, left, surface), and to the interacting residues in FtsW-FtsI (modeled based on 6PL5, right, surface). FtsB: blue; FtsL: pink; FtsQ: yellow; PBP1b: green; FtsW: cyan; FtsI: purple; CCD: green, Cα spheres; and AWI: purple, Cα spheres. The largest possible distance of the key residues in FtsWI was measured by flipping the periplasmic domain of FstI for 90° upwards. The regulatory relationship between them is shown below the molecules. **e** Crystal structures of the FtsB^E56A^LQ (light blue) and FtsB^D59H^LQ (blue) complexes superimposed with the FtsB^WT^LQ (dark blue), showing the displacement of the α2-α3 linker in FtsB. FtsL and FtsQ: white. **f** The cell length (left) and width (right) of the complemented cells by *ftsB*^WT^, *ftsB*^E56A^, or *ftsB*^D59H^. Median (shown on bottom) with interquartile range is shown. FtsB^WT^: *n* = 564; (FtsB^E56A^: *n* = 535, *P* < 0.0001 (length); FtsB^D59H^: *n* = 534, *P* < 0.0001 (width). **g–i** A hypothetical model for the regulation of PG synthases by FtsBLQ. **g** The mutation in the AWI domain leads to the impaired interaction and activation of FtsWI (dashed line of the activation arrow). **h** The mutation of FtsB^E56A^ and FtsB^D59H^ in the CCD domain may lead to a potential conformational change to enhance the activation on FtsWI (solid line of the activation arrow) and impair the inhibition on PBP1b (dashed line of the inhibition arrow), with an overall shift towards the activating state. **i** The recruitment of FtsN triggers constriction through FtsA and FtsBLQ and activates both FtsWI and PBP1b, which is mimicked by CCD mutations.

In the immediate vicinity of Site I, the interaction Site II harbors several key functional residues for FtsBLQ regulation (Fig. 4a). The tightly bent short helix of FtsB-α3 is sandwiched by the short helix of FtsL-α2 and the β domain of FtsQ, forming an extensive hydrogen-bond/electrostatic interaction network which involves the FtsB-FtsQ interface near the C-terminus of the coiled coil (Figs. 1c–e, 4b). Between the two short helices of FtsB and FtsL in Site II, hydrophobic residues occur periodically (*n* and *n* + 4) and face each other, including FtsB^L67, A71, L75^ and FtsL^V97, A101^ (Fig. 4b). On the outer surface of the two short helices, hydrophilic interactions dominate. On the FtsL side, FtsB^E74, R70^, and FtsL^H94^ bind together and connect with FtsB^E56^ and FtsL^E88^; on the FtsQ side, FtsQ^R196^ interacts with FtsB^E65, E69^, which further extends to involve residues of FtsB^E68, R72^ and FtsQ^R247, D245^ from Site I and residues of FtsB^E56, D59^ and FtsQ^R213^ from the lower part of Site II (Fig. 4b, Supplementary Table 2). Therein, FtsB^E56^ serves as an interaction hot spot, bringing together the β8-β9 and β10-α6 linkers of FtsQ, the α3 helix of FtsB, the α2 helix of FtsL, and the C-terminal region of the coiled coil (Fig. 4b). The importance of the FtsL-α2 helix was also highlighted by truncation mutants (FtsL^L100stop^ and FtsL^A90stop^) which exhibited a more severe growth defect in vivo than the FtsL^Q114stop^ mutant which had an intact α2^34^.

### Key functional domains of FtsBLQ for regulation of PG synthases

Two functionally critical domains are found to overlap with the interaction Site II of the FtsBLQ complex (Fig. 4c). One is the constriction control domain (CCD), involving approximately residues 88–94 in FtsL and 55–59 in FtsB^9^. Its disruption led to premature constriction probably by releasing the binding and repression of the TG domain of PBP1b by FtsBLQ^9,10^. The other is the activation of FtsW-FtsI domain (AWI), including R61, R82, N83, L84, L86, E87, and A90 of FtsL^11^. Mutations of the AWI residues exhibited a dominant-negative effect on cell division due to the impaired binding and activation of the FtsWI complex by FtsBLQ^11,12^. Several key findings about these domains emerge from the FtsBLQ complex structure. First, residues from both domains are located near the C-terminus of the ~49-Å-long FtsBL coiled coil (Fig. 1e, 4d), and the distance is too large if the protein is perpendicularly oriented, compared to the distance from their potential binding sites on PBP1b and FtsWI to the membrane plane (Fig. 4d). Yet when tilted, these domains can reach down (~28 Å) to interact with PBP1b-TG (up to ~25 Å) and FtsWI, either in the “down” (~25 Å) or “up” (~35 Å) conformation of FtsI based on the flipping model of its analog RodA-PBP2^36–38^ (Fig. 1f, 4d, Supplementary Fig. 9a, b).

Second, the structural proximity between the CCD and AWI domains in the FtsBLQ complex rules out the possibility of a simultaneous interaction with PBP1b and FtsWI (Fig. 4c). While it was known that mutual interactions occur between FtsBLQ, FtsWI and PBP1b, as tested by the co-purification^10^, and comparable affinities were observed for each pair (K_D_: 0.1-0.9 μM, Supplementary Fig. 9c) by using the bio-layer interferometry analysis in this study. A multi-step binding assay was also performed, showing an increased signal of PBP1b after the complex of FtsBLQ and FtsWI was already formed (Supplementary Fig. 9d). In this scenario, disrupting one of the interactions would not necessarily lead to the disassembly of the whole six-protein complex, while FtsBLQ may choose one of the two partners in an “either-or” manner. This finding echoes from a structural perspective with the previously proposed “on-and-off” model of FtsBLQ-mediated regulation^9^.

Finally, it is important to note that two CCD residues, FtsB^E56^ and FtsB^D59^, are buried in the FtsB-FtsQ interface, especially for the interaction hot-spot FtsB^E56^ which has an accessible surface area (ASA) of only 3.8 Å², about 2% in ASA ratio (Fig. 4b, c). In contrast, other CCD residues such as FtsL^E88^, FtsL^D93^ and FtsL^H94^ are surface accessible (ASA 51.2 Å², 71.6 Å², and 99.3 Å²), and some were implicated in the interaction with PBP1b (FtsL^D93^)^10^. We found that the mutations of FtsB^E56A^ or FtsB^D59H^ did not affect the complex formation on SEC (Supplementary Fig. 9e) or the overall structure (RMSD ~ 0.45 Å from the wild type) (Fig. 4e), and a mechanistic explanation is lacking for their apparent function in promoting cell constriction^9^. In our in vivo experiment, the strains carrying the complementing mutant plasmids also showed a reduction in the cell length (*ftsB*^E56A^) or cell width (*ftsB*^D59H^), similar to the previous report^9^ (Fig. 4f, Supplementary Fig. 7d). Herein, unless the FtsBLQ complex adopts a different conformation in vivo which exposes FtsB^E56^ and FtsB^D59^ for direct interactions with PBP1b or FtsWI, the only other possibility for these residues to affect cell division should lie in the FtsB-FtsQ interface, which connects the coiled coil to FtsQ (Fig. 1c–e). Mutations that disrupt this connection may detach the coiled coil from FtsQ, and the coiled coil may move accordingly to fit the short TM domains of FtsBL in a less tilted orientation in the membrane (Supplementary Fig. 2e). Subsequently, the position of all involving key regulatory residues would be altered, and interactions with different PG synthases could be modified in a way to increase the affinity with FtsWI and activate it, or in a way to decrease the affinity with PBP1b and release its inhibition, with a net outcome leading to the premature constriction (Fig. 4g, h).

Several experimental data are available to support this hypothesis. Crystal structures of FtsB^E56A^LQ and FtsB^D59H^LQ mutant complexes were solved, revealing a displacement of the FtsB α2-α3 linker for ~2–4 Å (Fig. 4e, Supplementary Table 1). While the overall structures remained almost the same probably due to the stabilization of crystal packing, the coiled coil might be more prone to move and this tendency could be transferred to their C-terminal linkers (Fig. 4e). When no such external force existed in solution, we observed a dramatic reduction of the higher-order oligomer band on native PAGE of the FtsB^E56A^ and FtsB^D59H^ mutants, indicating that the inter-oligomer contact may be affected by a potential allosteric change (Supplementary Fig. 4a). The observation may coincide with a previous study that the entire FtsBLQ was found to stimulate PG polymerization slightly less than FtsBL over time when complexed with FtsWI^12^, possibly due to the restrained flexibility of FtsBL by FtsQ. Although no conclusion could yet be made, the CCD residues, FtsB^E56^ and FtsB^D59^, are likely to function as a negative regulatory element to allosterically prevent any undesired movement in the balanced interplay between FtsBLQ and the PG synthases.

### An updated model of the FtsBLQ-mediated regulation

A hypothetical model of the FtsBLQ-mediated regulation is proposed based on the above findings. The “either-or” binding mode of FtsBLQ to the two PG synthases enables it to switch between two states, the FtsWI-binding (PG synthesis activated) and the PBP1b-binding (PG synthesis inhibited) states. Mutations that disrupt the interface with FtsWI (e.g., FtsL^E87K^) or PBP1b (e.g., FtsL^D93A^) can prevent its switch towards the FtsWI- (Fig. 4g) or the PBP1b-binding state^10–12^, thus delaying or promoting cell constriction, respectively. Mutations of FtsB^E56^ and FtsB^D59^, on the other hand, may induce a conformational change of the coiled coil to further enhance the activation of FtsWI, impair the inhibition of PBP1b and promote constriction (Fig. 4h). These mutations may somewhat mimic the effect of the FtsN arrival in the natural cell division process^9,12^. As FtsN is known to be recruited by the septal PG produced by these PG synthases, its arrival will induce a positive feedback loop to bind, sequester and activate PBP1b, thus releasing the FtsBLQ complex to interact with and activate FtsWI^8–11^, and in the meantime to act through FtsA and trigger the constriction of the septal ring (Fig. 4i). It will remain elusive whether the proposed conformational change of the FtsBL coiled coil could naturally occur until the seven-protein complex structure of FtsBLQ-FtsWI-PBP1b-FtsN becomes available one day.

## Discussion

In this study, we described the structure of a highly conserved, heterotrimeric membrane protein complex, FtsB-FtsL-FtsQ, as an essential component of the bacterial divisome. The three proteins form an inverted V-shape architecture with the two branches of different lengths joined at the membrane-distal tip. A tilted orientation for the membrane insertion of this complex is proposed, which provides spatial consistency with the position of key residues on its interactors, such as FtsK, FtsWI, and PBP1b. The 4-to-5-heptad long heterodimeric coiled coil of FtsBL was revealed to be slightly overwound and intrinsically flexible. Two extensive interaction sites were found to involve the C-terminal parts of all the three proteins, by forming a heterotrimeric β-sheet and holding the coiled coil close to the side of FtsQ. Biochemical, biophysical, and phenotypic assays were performed to study key interface residues, which led to a deeper understanding of their specific roles in the structure as well as in the FtsBLQ-mediated regulation during cell division. An allosteric change of the FtsBL coiled-coil domain is also proposed to explain the underlying mechanism of the control of constriction.

The tilted conformation of the FtsBLQ complex was unexpected and not reported before. However, there remain two possibilities that the complex could still fit in the membrane with perpendicular TM insertions. One is the domain rearrangement by disengaging the FtsB-FtsQ contact due to mutations or triggered by the interaction with other divisome proteins. Indeed, in the very recent cryo-EM structure of the FtsBLQ-FtsWI complex of *P. aeruginosa*^39^, the FtsW-FtsL interaction forces a perpendicular insertion of FtsBL, but the POTRA domain of FtsQ may be reoriented to fit the TM helix of FtsQ in the membrane. The other possibility is a curved membrane to have all the TM domains perpendicularly inserted. Membrane curvature is currently an intriguing topic in the bacterial divisome field, especially at the septal ring where the membrane must be curved for constriction to happen. Recently, the inner and outer membranes and the peptidoglycan layer have been visualized during bacterial cell division, showing the curvature and quick invagination of the inner membrane, yet without resolving any involved membrane proteins at the current resolution^40^. Due to the low-copy number of FtsB, FtsL, and FtsQ proteins in vivo, membrane curvature is less likely to be induced by this complex but may instead facilitate them to form a complex and perform its regulatory functions through allosteric changes. As the cryo-Electron Tomography (cryo-ET) technology has been rapidly advancing these years^41^, it will be exciting to uncover the conformation of the FtsBLQ complex embedded in the real or virtual lipid bilayer, and even see how the divisome regulation is realized via the dynamic domain/protein rearrangements, the direction of septal PG growth and the physical strength of a curved membrane.

The in vivo regulatory role of the interface residues of FtsBLQ was examined using the CRISPRi method. This approach allowed effective depletion of the *ftsB* gene for studying cell division phenotypes (Supplementary Fig. 6) and for subsequent rescue experiments by supplying extrachromosomal *ftsB* (Supplementary Fig. 7). Despite the unexpected polar effect on the downstream gene expression that attenuated cell viability (Supplementary Fig. 6a, c, f), the wild-type *ftsB* gene alone was sufficient to restore division and produce close to normal cells (Supplementary Fig. 7a–d). Together with the low-level expression, this approach provided a reliable basis for evaluating the *ftsB* mutants in cell division, thus allowing us to demonstrate that the FtsB^R72A^ mutation at the interaction site I and the FtsB^W20A^ mutation at the TM domain produced elongated cells, and the FtsB^E56A^ and FtsB^D59H^ mutations at the CCD domain influenced cell length or width, respectively. Therefore, while the results of the FtsB^R72A^, FtsB^E56A^, FtsB^D59H^ mutations are consistent with previous studies using the traditional technique^9,22^, this study also reveals the impact of the residue FtsB^W20^ that was not appreciated before (Fig. 2c, d). In the case of FtsB^W20A^, the inherent limitations of this and other methods^29^ may have caused the discrepancy.

In addition, although previous studies suggested that FtsBLQ could regulate the TG activity of PBP1b through the CCD domain as demonstrated by the FtsL^D93A^ mutant in vitro^10^, a genetic interaction between the two is still lacking. Furthermore, FtsW was found to inhibit PBP1b in the absence of FtsI in vitro^42^, and the interplay between these proteins may be more complicated than imagined based on our current understandings. As a non-essential PG synthase, PBP1b has a redundant homolog, PBP1a, and is thought to play a primary role in repairing cell wall defects but a minor role in the divisome^14,43^. On the other hand, PBP1b is also directly activated by FtsN for septal PG synthesis^10,13^. The current study could neither unravel this puzzle nor overlook the significance of PBP1b, which is potentially involved in FtsBLQ-mediated regulation. Further studies are in need of a full picture to be gradually unveiled, and the structure of the full-length FtsBLQ trimer would be an important piece for mechanistic inspirations from an allosteric perspective.

Finally, research in the field of bacterial divisome is expected to be ultimately contributing to the discovery of antibiotics with novel mechanisms. Divisome proteins FtsB, FtsL, and FtsQ are highly conserved, relatively easy to access, and lacking human homologs^1,2^, which make them ideal targets for the future development of more effective antibiotics that can be transferable to other Gram-negative bacteria with low side effects. A protein-protein interaction inhibitor, 17fα, has been recently published as an FtsB-derived proteomimetic that targets the FtsB-FtsQ interface^17^. The atomic structure of the FtsBLQ complex presented in this study, as well as more structures of similar complexes to be solved in the future, will become an indispensable asset to this field to inspire new targeting strategies for the non-catalytic proteins and divisome regulation.

## Methods

### Plasmid construction

The oligonucleotides, bacteria strains, and plasmids used in this study are summarized in Supplementary Tables 3 and 4. The construction of the plasmids reported in this study is described as below.

pRSF-FtsBL: the *ftsB*-*ftsL* sequence from *E. coli* strain K12 (ATCC10798D-5) was synthesized and purchased from Genomics BioSci & Tech. (Taiwan) (Supplementary Table 3). In order to enhance the expression level of the wild-type FtsL protein^20^, point mutations *ftsL*^S3N, R4K, V5L^ were introduced. Shine–Dalgarno (SD) sequence (AAGGAG) as the ribosome binding site is included. Synthesized sequence with restriction sites, *(Eco*RI*) - ftsB - (Sal*I*) -* SD *- ftsL- (Hin*dIII), was inserted into the pRSFDuet^TM^−1 vector.

pRSF-FtsBLQ: *ftsQ* was amplified from the genome of *E. coli* strain K12 (ATCC10798D-5) by primers Q-F and Q-R, and inserted into the pRSFduet-BL plasmid at *Hind*III/*Not*I sites, generating the construct of (*Eco*RI*) - ftsB - (Sal*I*) - SDtag - ftsL- (Hin*dIII) – SDtag - *ftsQ – Not*I in the pRSFDuet^TM^−1 vector.

pRSF-FtsBLQ+Thrombin: Thrombin cutting site was inserted into the pRSF-FtsBLQ plasmid, after the N-terminal His-tag. One step overlapped extension PCR using BLQ + Thrombin-F and BLQ + Thrombin-R primers were performed to insert the Thrombin cutting site, generating the pRSF-FtsBLQ+Thrombin plasmid.

pET15b-PBP1b was reported previously^38^, in which the gene fragment encoding PBP1b (aa58-804) from the *E. coli* strain K12 (ATCC10798D-5) was inserted into pET15b at *Nde*I/*BamH*I.

pETduet-FtsWI: *ftsW* and *ftsI* were amplified from the genome of *E. coli* strain K12 (ATCC10798D-5) by primers W-F and W-R, I-F, and I-R, respectively. Amplified *ftsW* was inserted into the multicloning site I at *BamH*I/*EcoR*I, and amplified *ftsI* was inserted into the multicloning site II at *Aat*II/*Xho*I of the pETduet-1 vector.

The CRISPR interference (CRISPRi) system employs a plasmid pFD152 that carries the *dCas9* gene under the control of the *P*_*tet*_ promoter^44^. pSOT357 was generated by ligating the guide RNA, sgRNA*ftsB* (Supplementary Fig. 6a, Supplementary Table 3), after the constitutive promoter *P*_*pflB*_ in pFD152.

The complementation plasmids were created by placing the wild-type or mutant *ftsB* gene under the control of the *P*_*lac*_ promoter in pMLB1113^45^. pSOT359 (*P*_*lac*_*-ftsB*^*WT*^) carried the *ftsB* gene that was PCR amplified from the chromosome, digested with *EcoR*I and *Hind*III, and ligated in pMLB1113. pSOT362 (*P*_*lac*_*-ftsB*^*Q16A*^), pSOT363 (*P*_*lac*_*-ftsB*^*W20A*^), and pSOT364 (*P*_*lac*_*-ftsB*^*Q16A-W20A*^), pSOT388 (*P*_*lac*_*-ftsB*E56A), and pSOT360 (*P*_*lac*_*-ftsB*^*D59H*^) were created by ligating the mutant *ftsB* that was PCR amplified from pSOT358 (*P*_*ftsB*_*-ftsB*^*Q16A*^), pSOT355 (*P*_*ftsB*_*-ftsB*^*W20A*^), pSOT356 (*P*_*ftsB*_*-ftsB*^*Q16A-W20A*^), pRSF-*ftsB*^*E56A*^*LQ*, and pRSF-*ftsB*^*D59H*^*LQ*, respectively. In the PCR reactions, the RBS sequence and *EcoR*I and *Hind*III restriction sites were introduced in primers (Supplementary Table 3). The plasmids, pSOT358, pSOT355, and pSOT356, were created by overlapping PCR to introduce the mutation (Q16A, W20A, or double mutant Q16A-W20A) in *ftsB* that was then cloned in pKD3 at the *Sal*I and *BamH*I sites.

The plasmid pSOT414 (*P*_*lac*_*-ftsB*^*R72A*^) was generated by Gibson Assembly (New England Biolabs, Inc.). The gene fragment of *ftsB*^*R72A*^ was PCR amplified in two reactions to introduce the point mutation along with the RBS sequence. The two PCR fragments and the linearized pMLB1113 were ligated in a Gibson Assembly reaction.

All other mutants in this study were generated by site-directed mutagenesis using KOD Hot Start DNA Polymerase (Sigma) and then were confirmed by DNA sequencing.

### Protein expression and purification

The FtsBLQ complex, the FtsW-FtsI complex, the PBP1b (aa58-804) protein, and all related mutants were purified as previously described^10,38^ with modifications as below. Proteins were overexpressed in *E. coli* C43 strain (Sigma). Single colonies of fresh transformants were inoculated for growing overnight at 37 °C in TB (Terrific Broth) medium supplemented with appropriate antibiotics (ampicillin 100 μg/ml or kanamycin 50 μg/ml). When the OD_600nm_ reached 0.6, 1 mM isopropyl β-D-1-thiogalactopyranoside (IPTG) was added and the culture continued to grow at 37 °C for 4 hours. Cells were harvested, clarified with centrifugation at 9000 × *g* (JLA 8.1000 rotor, Beckman) for 10 min, and frozen at −80 °C. Cells were thawed and fully resuspended in lysis buffer (20 mM Tris-HCl pH8.0, 200 mM NaCl, 20 mM imidazole, 10 μg/ml DNase I, 1 mM PMSF and one tablet of the complete EDTA-free protease inhibitor cocktail, Roche) before they were passed through the microfluidizer three times. After being centrifuged at 27,000 × *g* (JA 25.50 rotor, Beckman) at 4 °C for 30 mins, the pellet was collected and resuspended in the solubilization buffer (20 mM Tris-HCl pH8.0, 200 mM NaCl, 10% glycerol, EDTA-free protease inhibitor cocktail), and a final concentration of 2% 5-Cyclohexyl-1-Pentyl-β-D-Maltoside (Cymal-5, for structural studies) or 1% n-Dodecyl-β-D-Maltoside (DDM, for kinetic studies) was added, and the mixture was moderately stirred at 4 °C for 4 hours. All detergents were purchased from Anatrace. The solubilized sample was clarified by centrifugation at 27,000 × *g* (JA 25.50 rotor, Beckman) for 30 mins at 4 °C, and the supernatant was loaded onto a Ni-NTA gravity column (GE healthcare) and washed extensively by the buffer containing 20 mM Tris-HCl pH8.0, 200 mM NaCl, 0.2% Cymal-5 and 40 mM imidazole. The bound protein was either eluted by the same buffer supplemented with 500 mM imidazole, or digested by Thrombin (Sigma) at the cutting site after the N-terminal His-tag at room temperature overnight, depending on the purpose of each experimental design. The digested protein was then eluted with the same buffer supplemented with 20 mM imidazole. The pure eluted fractions were combined and further purified by the Superdex-200 10/300 GL column (Cytiva) in either the Cymal-5 buffer (200 mM NaCl, 20 mM HEPES pH7.5, 0.2% Cymal-5) for crystallization purpose or the DDM buffer (200 mM NaCl, 20 mM HEPES pH7.5, 0.1% DDM) for Bio-Layer Interferometry assay.

### Crystallization, data collection and processing

The purified FtsBLQ, FtsB^D59H^LQ, and FtsB^E56A^LQ complexes with the His-tag were concentrated using an Amicon centrifugal concentrator with a 50 kDa molecular weight cut off (Merck) to 10 mg/ml in 20 mM HEPES pH7.5, 200 mM NaCl, 0.2% Cymal-5 and used to set up crystallization in the sitting-drop vapor diffusion method at 20 °C. Protein was mixed with mother liquid (ratio 1:1). Crystals appeared after 1-3 days in the condition of 0.1 M Tris-HCl pH8.0, 20-35% PEG400, which then were harvested and flash cooled in liquid nitrogen for data collection. X-ray diffraction datasets were remotely collected at TPS 05A beamline of National Synchrotron Radiation Research Center, Taiwan or at X06SA-PXI beamline of Swiss Light Sources at Paul Scherrer Institut, Switzerland. Collected data were indexed, integrated, and scaled using iMosflm ver 7.2.2^46^ and Aimless in CCP4i ver 7.0.063 for the wild-type and the FtsB^E56A^LQ mutant. For FtsB^D59H^LQ mutant, data were processed by XDS BUILD = 20190806^47^ and four datasets were merged by XSCALE BUILD = 20190806^47^. Three FtsBLQ complex structures (FtsBLQ, FtsB^D59H^LQ, and FtsB^E56A^LQ) were determined at a final resolution of 3.1 Å, 3.0 Å, and 3.3 Å, respectively.

### Phasing, model building and structural refinement

The structure of FtsQ in complex with the C-terminal peptide of FtsB (PDB: 5Z2W^18^) was used for molecular replacement (MR) in Phenix phaser ver 1.14-3260 and the MR solution was found with a TFZ of 14.8, one molecule copy per asymmetric unit (ASU) and a bulk- solvent content of over 80%. We started from this MR solution to manually build the TM of FtsQ, the rest of FtsB, and the entire FtsL structure (~40% atoms of the final model) in WinCoot ver 0.8.9^48^. The density of the long coiled-coil FtsBL was clear at the beginning of model building (Supplementary Fig. 8b). The residue register of FtsL was determined by the unambiguous density of the bulky side chains of W81 near the C-terminal end of the coiled coil and F43 in the transmembrane (TM) helix, further confirmed by the density of two histidine, H58, and H59 to define on the boundary between the TM and periplasmic parts. The TM region of FtsB contains several aromatic residues including W14, Y16, W20, F21, and H27, and the TM region of FtsQ contains W43 and W48, which all facilitated structural building. Residues on the coiled-coil part of FtsB were confidently defined based on the clear negative density at E56 when the WT structure was used for molecular replacement of the E56A mutant (Supplementary Fig. 8d). Hundreds of iterations of manual adjustments and structural refinements using WinCoot ver 0.8.9^48^ and Phenix.refine ver 1.14-3260^49^ eventually resulted in the converged R factors and revealed clear densities for all the rest of the structure (Supplementary Fig. 8b). Structural figures were prepared with PyMOL ver 1.3^50^.

### SEC-MALS

Multi-angle light scattering coupled with size exclusion chromatography of the purified FtsBLQ complex (wild-type or mutants) was performed by running in Superdex 200 GL 10/300 using the Cymal-5 buffer (200 mM NaCl, 20 mM HEPES pH7.5, 0.2% Cymal-5) on an Akta FPLC (GE healthcare) connected to a three-angle light-scattering detector (mini-DAWN TREOS) and a refractive index detector (Optilab T-rEX, Wyatt Technology, Santa Barbara, California). Data analysis was performed with ASTRA ver 6.0.5.3.

### Bio-Layer Interferometry

The FtsBLQ complex, the FtsWI complex, and PBP1b (aa58-804) protein with or without the His-tag were purified as mentioned above. Three assays were performed to analyze the binding affinity of PBP1b to His-tagged FtsBLQ, FtsBLQ to His-tagged FtsWI, and PBP1b to His-tagged FtsWI. All purified His-tagged proteins were loaded at 50 μg/ml in the kinetics buffer (200 mM NaCl, 20 mM HEPES pH7.5, 0.1% DDM) onto the HIS1K (Anti-Penta-HIS) biosensors (Molecular Devices, ForteBio) using the Octet HTX system (FroteBio). Association and dissociation kinetic curves were recorded for 240 sec each step, with the protein analytes in a threefold serial dilution from the highest concentration of 4 µM (range 0.016 µM − 4 µM). K_D_ values were calculated using the Octet Analysis software ver 9.0.0.10 for each biological replicate.

For the sequential binding assay, the His-tagged FtsBLQ was loaded at 50 μg/ml in the kinetics buffer (200 mM NaCl, 20 mM HEPES pH7.5, 0.1% DDM) onto the HIS1K (Anti-Penta-HIS) biosensors (Molecular Devices, ForteBio) using the Octet HTX system (FroteBio). Association 1 is FtsWI protein (4 µM) and Association 2 is the PBP1b protein (4 µM) then followed by the dissociation. Association and dissociation kinetic curves were recorded for 240 sec each step. The kinetics buffer alone was used as control.

### Native PAGE

The FtsBLQ complex, wild-type, and mutants, after being purified by the Superdex-200 column in the Cymal-5 buffer (200 mM NaCl, 20 mM HEPES pH8.0, 0.2% Cymal-5), were loaded onto the native gel electrophoresis using the precast NativePAGE™ 4-16%, Bis-Tris, 1.0 mm, Mini Protein Gels (Invitrogen™). Protein samples were prepared in the NativePAGE® Sample Buffer (4X), with the additive of NativePAGE® 5% G-250. The protein marker NativeMark™ Unstained Protein Standard (Invitrogen™) was used for molecular weight estimation. The chamber was filled with 1X NativePAGE® Cathode Buffer (upper) and 1X NativePAGE® Anode Buffer (lower). The NativePAGE gel was run at a voltage of 150 V for 120 minutes on ice. The gel was fixed by 40% methanol, 10% acetic acid for 45 s, destained by 8% acetic acid for 45 s with heating in a microwave, according to the manufacturer’s instructions, and then shaken in water at room temperature until the desired background is obtained.

### Biological Small-Angle X-ray Scattering (Bio SAXS)

The purified protein FtsBLQ with the unremoved His-tag at the concentration of 10 mg/ml in the buffer containing 20 mM HEPES pH7.5, 200 mM NaCl, 0.2% of Cymal-5 were used for SAXS measurement. SAXS data were collected at beamline TPS 13A BioSAXS of the National Synchrotron Radiation Research Center, Taiwan^51,52^. The SEC-SAXS system included a focused X-ray beam, in-vacuum Eiger X 9M and X 1M detectors coupled with an in-line HPLC unit (Agilent 1260 series), and a Bio SEC-3 silica-based column (pore size 300 Å, Agilent). The column was equilibrated with the buffer first, then 100 μl of FtsBLQ at 10 mg/ml was loaded onto the column with a flow rate of 0.35 ml/min at 10 °C. The eluate from the SEC run was directed to the SAXS system. The scattering data were collected with a frame rate of 2 s per frame (0.2 s between frames) over the elution peak. The frames with a close radius of gyration were averaged and used for data analysis. Data were background subtracted and analyzed using NSRRC TPS 13A SAXS Data Reduction Kit (Ver. 4.79) and ATSAS 3.2.1 package^53^. Specifically, the X-ray scattering curves of the trimeric crystal structure and the potential hexamer models were calculated using the CRYSOL program^53^. Ten independent models were generated using DAMMIN software^53^, then averaged by DAMAVER to reconstruct the ab initio envelope of the FtsBLQ complex. The averaged model was visualized and converted to the surface mesh of the equivalent electron density map at a resolution of 15 Å in UCSF Chimera ver 1.16^54^. Two trimeric crystal structures were then fit in manually. Structure figures were generated using UCSF Chimera 1.16^54^.

### *ftsB* knock-down using CRISPRi

The *E. coli* strain MC1000^55^ was used in the *ftsB* knock-down experiment. The guide RNA (Supplementary Fig. 6a and Table 3) was selected using the online tool ‘Guide RNA design for CRISPRi in bacteria’ in the CRISPR browser @ Pasteur^56^ (https://crispr-browser.pasteur.cloud/). The off-target sites of the predicted guide RNAs were searched on the *E. coli* K12 substr. MG1655 genome using the sequence pattern search tool, ‘PatMatch’, in the EcoCyc database^57^ (https://ecocyc.org/). The sgRNA*ftsB* reported in this study has an advantage for the subsequent complementation experiment, although it showed a lower score of −0.28 in the CRISPR prediction. Owing to its location at the promoter region of *ftsB*, it was not necessary to change the codons that match to the sgRNA*ftsB* priming site in *ftsB* on the complementing plasmid. In addition, this sgRNA is predicted to have only one off-target site in a non-essential gene *ydhC*. In addition, there are 9 potential bad-seed sites predicted, but only *holA* is an essential gene. Nonetheless, since we need only 0.05 µg/mL anhydrotetracycline (aTc) to induce dCas9 expression and effectively knock-down the *ftsB* expression, there is no good reason to worry about the bad-seed effect in our experiments (Supplementary Fig. 6f). Thus, neither the off-target nor the bad-seed effect had significant influence to our observations. Meanwhile, the polar effect on the downstream genes of *ftsB* was seen unexpectedly on the *ispD-ispF*. Nonetheless, the cell division phenotype was effectively rescued in the knock-down strain to permit examination of the mutant *ftsB* in cell division (Supplementary Fig. 7d-e).

The strain MC1000 carrying empty vector pFD152 or pSOT357 was grown from a single colony in the lysogeny broth (LB) supplemented with 50 µg/mL spectinomycin (Spe) overnight at 30 °C. The overnight culture was diluted to OD_600nm_ 0.05 in fresh LB containing 50 μg/mL Spe alongside with 0, 0.025 μg/mL, 0.05 μg/mL, or 0.1 μg/mL aTc to induce the dCas9 expression. The cultures were monitored for cell growth. In addition, cells were collected at OD_600nm_ 0.6 (around 3.5 hours) and fixed with 0.2% glutaraldehyde and 2% formaldehyde for microscopy. Judging from results, the induction condition of 0.05 μg/mL aTc was chosen for the complementation experiments.

### Complementation assay

The complementing plasmid carry wild-type or mutant *ftsB* (Supplementary Table 4) was co-transformed with pSOT357 into MC1000. The strain was grown from a single colony and cultured overnight at 30 °C in LB supplemented with 50 μg/mL Spe, 50 μg/mL ampicillin (Amp), and 0.4% glucose (Glc). In the next day, the culture was refreshed in the same medium containing 0.05 μg/mL aTc until OD_600nm_ reached 0.4~0.5. Cells were washed and resuspended in the fresh same medium, but 0.4% Glc, no suppressor/inducer (Nil), or 15 µM isopropyl β-D-1-thiogalactopyranoside (IPTG) was added in independent cultures to repress or induce complementing gene expression. The cultures were incubated for 3 hours at 30^o^C (OD_600nm_ 0.397–0.637) before harvesting cells for RT-qPCR or for microscopy by fixation with 0.2% glutaraldehyde and 2% formaldehyde for 20 min at room temperature. The optimal condition of complementation was determined as without inducer. This basal-level expression showed only one-fold increase in comparison with the endogenous level, and sufficient for *ftsB*^WT^ to rescue the division phenotype (Supplementary Fig. 7b–d).

### RT-qPCR

Total RNA was extracted from cells collected from the knock-down and complementation experiments using the Bacteria RNA Mini Kit with DNase (Geneaid Biotech Ltd., New Taipei, Taiwan). The complementary DNA (cDNA) was prepared from the total RNA by reverse transcription using the Invitrogen™ SuperScript™ III First-Strand Synthesis SuperMix (cat. 18080400, Thermo Fisher Scientific Inc., Waltham, MA USA). Relative quantitation of cDNA was performed using the LightCycler® 480 SYBR Green I Master kit (Roche Molecular Systems, Inc., Pleasanton, CA, USA) and the amplification cycles were measured on LightCycler® 480 Instrument (Roche Molecular Systems, Inc. Pleasanton, CA, USA). The constitutively expressed *cysG* was used as the reference gene in our study based on Zhao et al, (2011)^58^. The primer pairs, including *cysG*-F and *cysG*-R, *ftsB*-F and *ftsB*-R, *ispD*-F and *ispD*-R, and *ispF*-F and *ispF*-R, were used for RT-qPCR experiment (Supplementary Table 3). The experimental steps were operated according to the qPCR protocol published in Mückl et al. (2018)^59^ as well as the procedures recommended in the instruction manuals of the kits.

The relative expression ratio between the target (*ftsB*, *ispD*, and *ispF*) and reference (*cysG*) genes was calculated following the mathematical model for relative quantification in real-time PCR as described in Pfaffl (2001)^60^. The ratios of the *ftsB* mutants were then normalized with the ratios of the wild-type *ftsB* under each condition (0.4% Glc, Nil, 15 µM IPTG).

### Microscopy

Cells collected after *ftsB*- knock-down and complementation were centrifuged, resuspended in PBS (10 mM Na_2_HPO_4_, 1.8 mM KH_2_PO_4_, 137 mM NaCl, 2.7 mM KCl, pH7.4). The cell suspension was stained with the lipophilic dye FM4-64 (Invitrogen^TM^) at 1 µg/mL and the DNA dye 4,6-diamidino-2-phenylindole (DAPI) (Sigma) at 1 µg/mL, and incubated in dark for 20 min. Cells were then washed with PBS by pelleting at 6000 × *g* for 2 min followed by resuspension. Cell fixation was performed with 2% formaldehyde and 0.2 % glutaraldehyde for 20 min at room temperature. Cells were then washed three times with PBS by pelleting at 6000 × *g* for 2 min followed by resuspension. Stained and fixed cells were visualized by placing onto 2% PBS agarose pads.

Phase contrast and fluorescence microscopy were performed on an Olympus IX81 inverted microscope (Olympus, Japan) equipped with a CCD camera (ORCA-R2, Hamamatsu, Japan), an objective lens (UPlanFLN 100x, NA 1.30, Olympus, Tokyo, Japan), and filter sets for DAPI (Chroma cat. 89402, Multi LED set) and FM4-64 (Chroma cat. 11007v2 - Wide Green). Images were acquired using cellSens Dimension (2019) (Olympus Corp., Tokyo, Japan) with an X-Cite® TURBO multiwavelength LED illumination system (Excelitas Technologies Corp., Ontario, Canada). The phase-contrast images were analyzed for length and width using the MicrobeJ 5.11j plugin^61^ installed in NIH ImageJ ver 1.53a (Rasband, W.S., ImageJ, U.S. National Institutes of Health, Bethesda, Maryland, USA, http://rsb.info.nih.gov/ij/). The lysed cells, that may be produced after *ftsB* depletion for 3 hours before inducing the complementing *ftsB* as well as the attenuated expression of the downstream genes after *ftsB* knock-down, were identified as phase-light objects and were excluded from the measurements.

### LC-MS/MS

Samples were detected by LC-ESI-MS on a Orbitrap Fusion mass spectrometer (Thermo Fisher Scientific, San Jose, CA) equipped with EASY-nLC 1200 system (Thermo, San Jose, CA, US) and EASY-spray source (Thermo, San Jose, CA, US). The digestion solution was injected (5 μl) at 1 μl/min flow rate on to easy column (C18, 0.075 mm × 150 mm, ID 3 μm; Thermo Scientific) Chromatographic separation was using 0.1% formic acid in water as mobile phase A and 0.1% formic acid in 80% acetonitrile as mobile phase B operated at 300 nl/min flow rate. Briefly, the gradient employed was 5% buffer B at 2 min to 40% buffer B at 50 min. Full-scan MS condition: mass range m/z 375–1500 (AGC target 5E5) with lock mass, resolution 60,000 at m/z 200, and maximum injection time of 50 ms. The MSMS was run in top speed mode with 3 s cycles with CID; while the dynamic exclusion duration was set to 60 s with a 10 ppm tolerance around the selected precursor and its isotopes. Electrospray voltage was maintained at 1.8 kV and capillary temperature was set at 275 °C.

### Statistical analysis

Statistical analyses were performed using GraphPad Prism ver 9.1.0 (GraphPad Software, Inc., USA). Significant differences between different conditions were determined either by Ordinary one-way ANOVA (Tukey’s multiple comparisons test) or two-tailed *t*-test. ****P < 0.0001, ***P < 0.001, **P < 0.01, *P < 0.05.

### Reporting summary

Further information on research design is available in the Nature Portfolio Reporting Summary linked to this article.

## Supplementary information

Supplementary Information

Reporting Summary

## Data Availability

The data that support this study are available from the corresponding authors upon request. The coordinates of the refined models have been deposited into the Protein Data Bank (PDB) with the accession codes 8HHG (FtsBLQ), 8HHF (FtsB^D59H^LQ), and 8HHH (FtsB^E56A^LQ). Previously published PDB codes that referred in this study are also available in the Protein Data Bank, including 5Z2W, 2VH1, 6H9N, 5HLB, 6PL5. All other data generated in this study are present in the paper, the supplementary information file, and the source data file. Source data are provided with this paper.

## Source data

Source Data
