# Supplementary material for: Structure of the heterotrimeric membrane protein complex FtsB-FtsL-FtsQ of the bacterial divisome
Source: Nat Commun. 2023 Apr 5;14:1903. doi: 10.1038/s41467-023-37543-4 (PMC10076392; doi:10.1038/s41467-023-37543-4)

SUPPLEMENTARY INFORMATION

Structure of the heterotrimeric membrane protein complex FtsB-FtsL-FtsQ of the bacterial divisome

Hong Thuy Vy Nguyen[1,2,3,8], Xiaorui Chen[1,8], Claudia Parada[4], An-Chi Luo[3,4], Orion Shih[5], U-Ser Jeng[5,6], Chia-Ying Huang[7], Yu-Ling Shih[3,4*], Che Ma[1*]

[1] Genomics Research Center, Academia Sinica, Taipei 115, Taiwan

[2] Chemical Biology and Molecular Biophysics program, Taiwan International Graduate Program, Academia Sinica, Taipei 115, Taiwan

[3] Institute of Biochemical Sciences, National Taiwan University, Taipei 10617, Taiwan

[4] Institute of Biological Chemistry, Academia Sinica, Taipei 115, Taiwan

[5] National Synchrotron Radiation Research Center, Hsinchu 30076, Taiwan

[6] Department of Chemical Engineering, National Tsing Hua University, Hsinchu 30044, Taiwan

[7] Paul Scherrer Institute, Forschungsstrasse 111, Villigen-PSI, 5232, Switzerland

* Corresponding author: e-mail: ylshih10@gate.sinica.edu.tw (Y.L.S.) and cma@gate.sinica.edu.tw (C.M.)

[8] These authors contributed equally to this work: Hong Thuy Vy Nguyen, Xiaorui Chen.

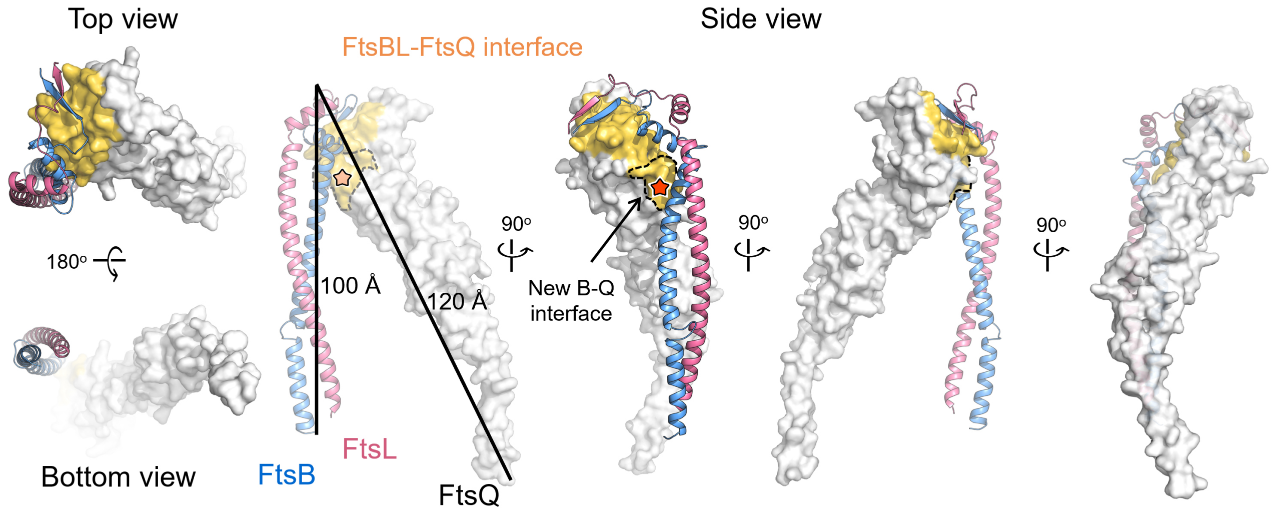

Supplementary Fig. 1: The FtsB-FtsL coiled coil drapes along FtsQ in the heterotrimeric complex.

Top, bottom and side views of the FtsBLQ complex showing FtsB (ribbon), FtsL (ribbon) and FtsQ (surface) colored in blue, pink, and white respectively. The interface between them is colored in yellow on FtsQ, with newly defined FtsB-FtsQ interface is highlighted with an orange star. The straight length of each branch of the V-shaped complex structure is also shown.

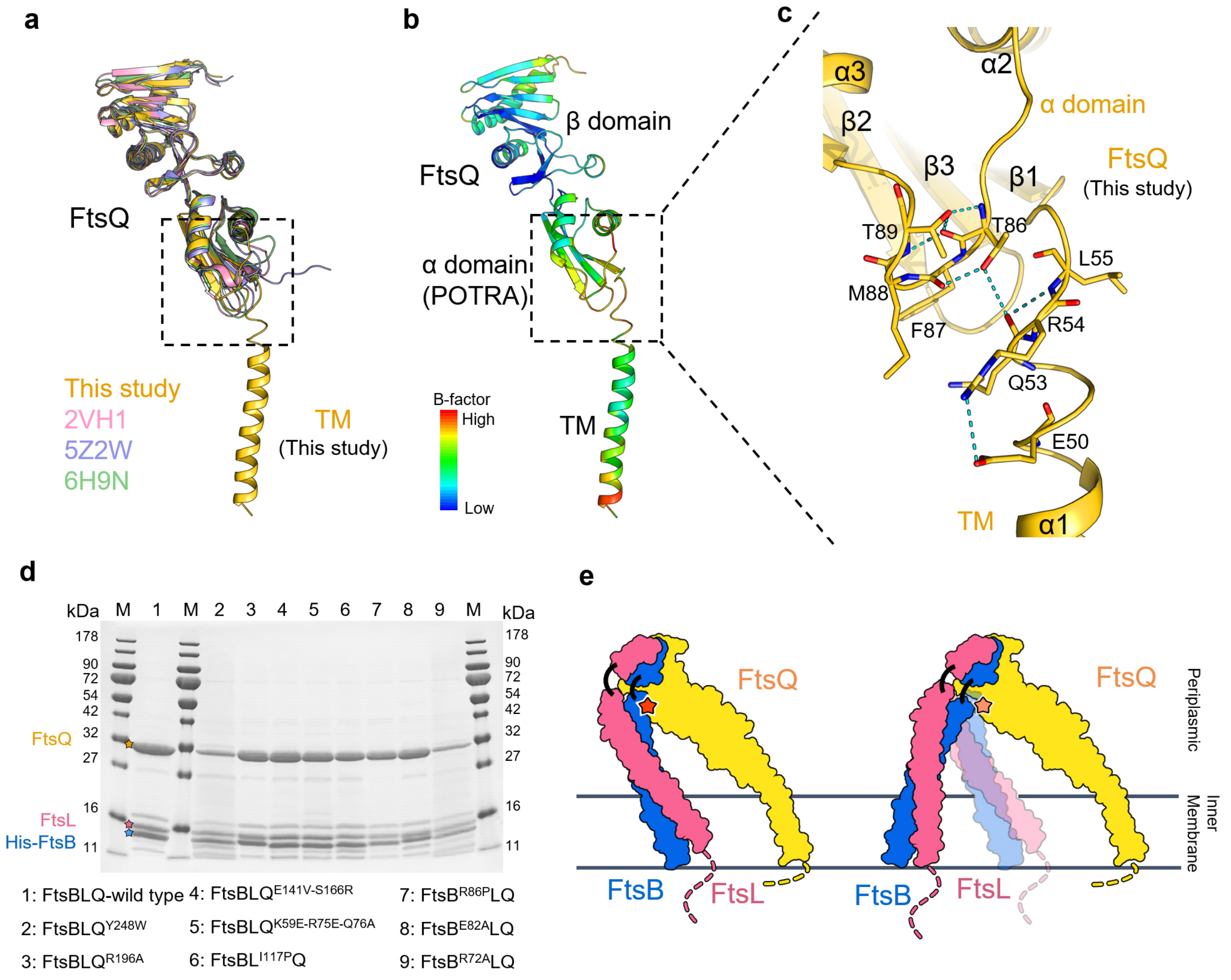

Supplementary Fig. 2: Structural analysis of FtsQ and mutagenesis studies.

a, The superimposition of the full-length FtsQ structure (this study, yellow) with previously published FtsQ structures without the TM domain (PDB: 2VH1 in light pink, 5Z2W in light purple, 6H9N in light green). The region with highest flexibility is highlighted in a box. **b**, The full-length FtsQ structure colored by B-factors in the same view as in (**a**). Red indicates the higher B-factors and blue the lower. **c**, An enlarged view of the boxed region in (**a**) and (**b**) showing interactions between FtsQ-TM and the periplasmic α domain involving α2-α3 linker, β2-β3 linker and TM-β1 linker. **d**, The representative raw SDS-PAGE gel image of the co-purification of His-FtsB, FtsL and FtsQ for both the wild-type and mutants in each lane as listed. Corresponding bands of His-FtsB, FtsL and FtsQ are labeled with blue, pink, yellow stars respectively. The middle bands are: the partially degraded FtsQ (~27kD), the undissociated and partially degraded FtsBL (~18kD) as determined by LC-MS/MS. This experiment was done with three independent replicates that showed similar results. **e**, The cartoon model of the FtsBLQ complex inserted into the membrane in the tilted orientation (left) and the FtsBLQ complex with a possible domain rearrangement (right) disengaging the new interface (orange star). FtsB: blue. FtsL: pink. FtsQ: yellow.

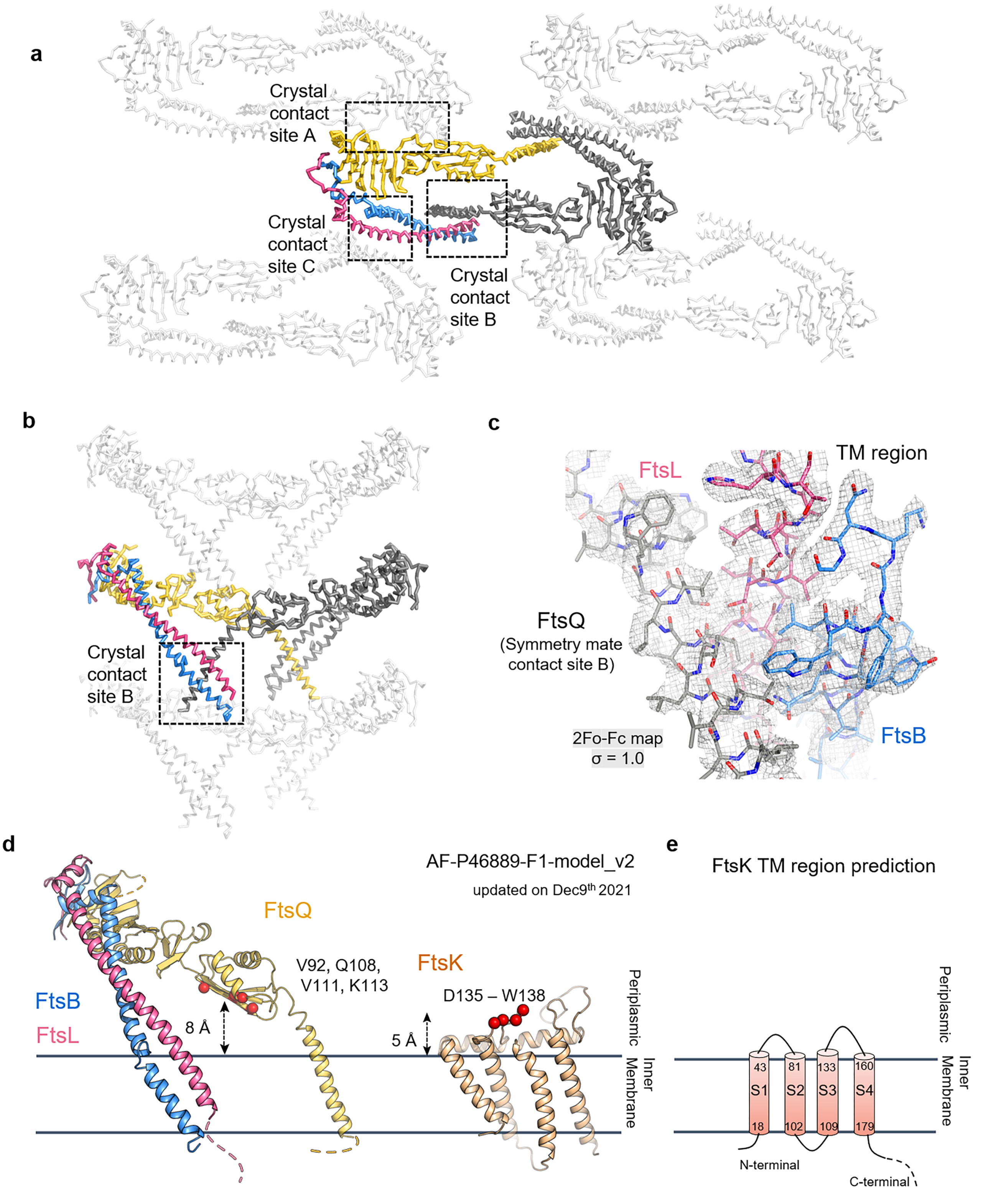

Supplementary Fig. 3: Crystal packing of the FtsBLQ complex and the spatial compatibility with its recruiter FtsK.

a, Crystal packing of the FtsB-FtsL-FtsQ complex reveals three inter-asymmetric-unit contact sites. FtsB: blue. FtsL: pink. FtsQ: yellow. The symmetry mate of contact site B is colored in dark grey

for highlighting. Other symmetry mates are colored in light grey. **b**, Side view of crystal packing relative to (**a**). **c**, The electron density map of the transmembrane helices of FtsB, FtsL and FtsQ (crystal site B as in **a**, **b**). **d**, Distances measured from the membrane plane to the potential interacting residues of FtsQ (yellow) in the same orientation as (**b**), and to the potential interacting residues of FtsK (wheat). Residues are shown as red Cα spheres. The N-terminal TM domain of FtsK was predicted by AlphaFold Monomerv2.0, updated on Dec 9th, 2021. **e,** The topology of FtsK-TM domain in secondary structural prediction. Cylinders indicate TM helices. The N- and C-termini and the start and end of each TM helix are labeled.

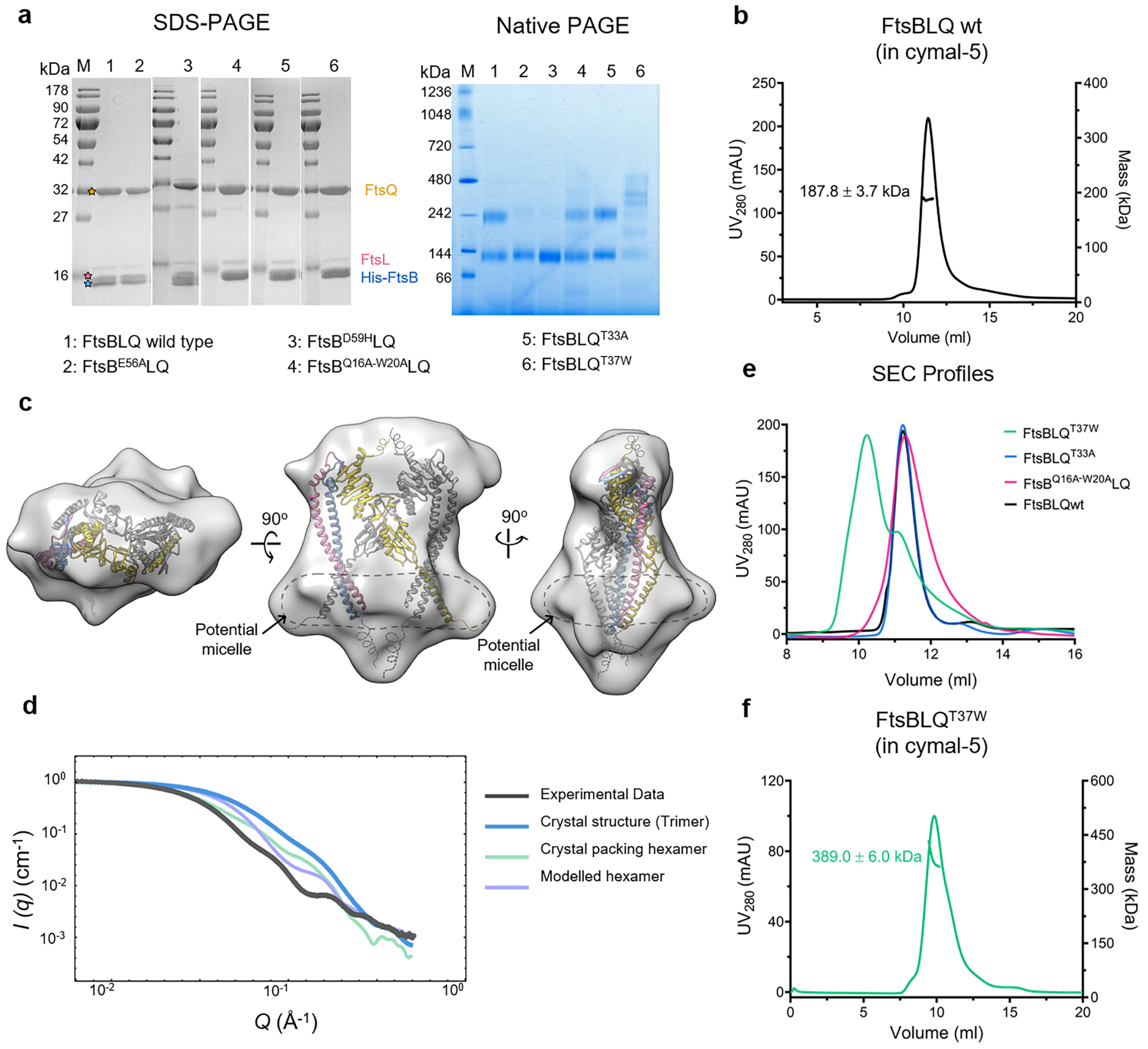

Supplementary Fig. 4: Possibility of FtsB-FtsL-FtsQ hexameric conformation.

a, The representative SDS-PAGE (left) and native PAGE (right) results of the FtsBLQ complex for both wild-type and mutants as listed. FtsB, FtsL, FtsQ corresponding bands are labeled with blue, pink, yellow stars, respectively. This experiment was done with three independent replicates that showed similar results. **b**, The SEC-MALS profile of the purified FtsBLQ wild-type complex in detergent solution (Cymal-5). The MALS trace (short line) on top of the SEC curve indicates a molecular mass of 187.8 ± 3.7 kDa. **c**, The averaged *ab initio* envelope calculated from SAXS for the FtsBLQ complex in different views, with two trimeric crystal structures of FtsBLQ (ribbon) manually modelled into the envelop. The potential space for micelles is labeled. **d**, The SAXS curves calculated from the crystal trimeric structure (blue), the crystal-packing hexamer (green) and the modelled hexamer as in **c** (purple), fit with the SAXS experimental data (black). **e**, SEC profiles

of the FtsBLQ complex for wild-type and different TM mutants. $FtsBLQ^{T37W}$: green, $FtsBLQ^{T33A}$: pink, $FtsB^{Q16A-W20A}LQ$: blue, FtsBLQ wild-type: black. **f**, The SEC-MALS profile of the purified $FtsBLQ^{T37W}$ mutant in detergent solution (Cymal-5). The MALS trace (short line) on top of the SEC curve indicates an estimated molecular mass of 389.0 ± 6.0 kDa .

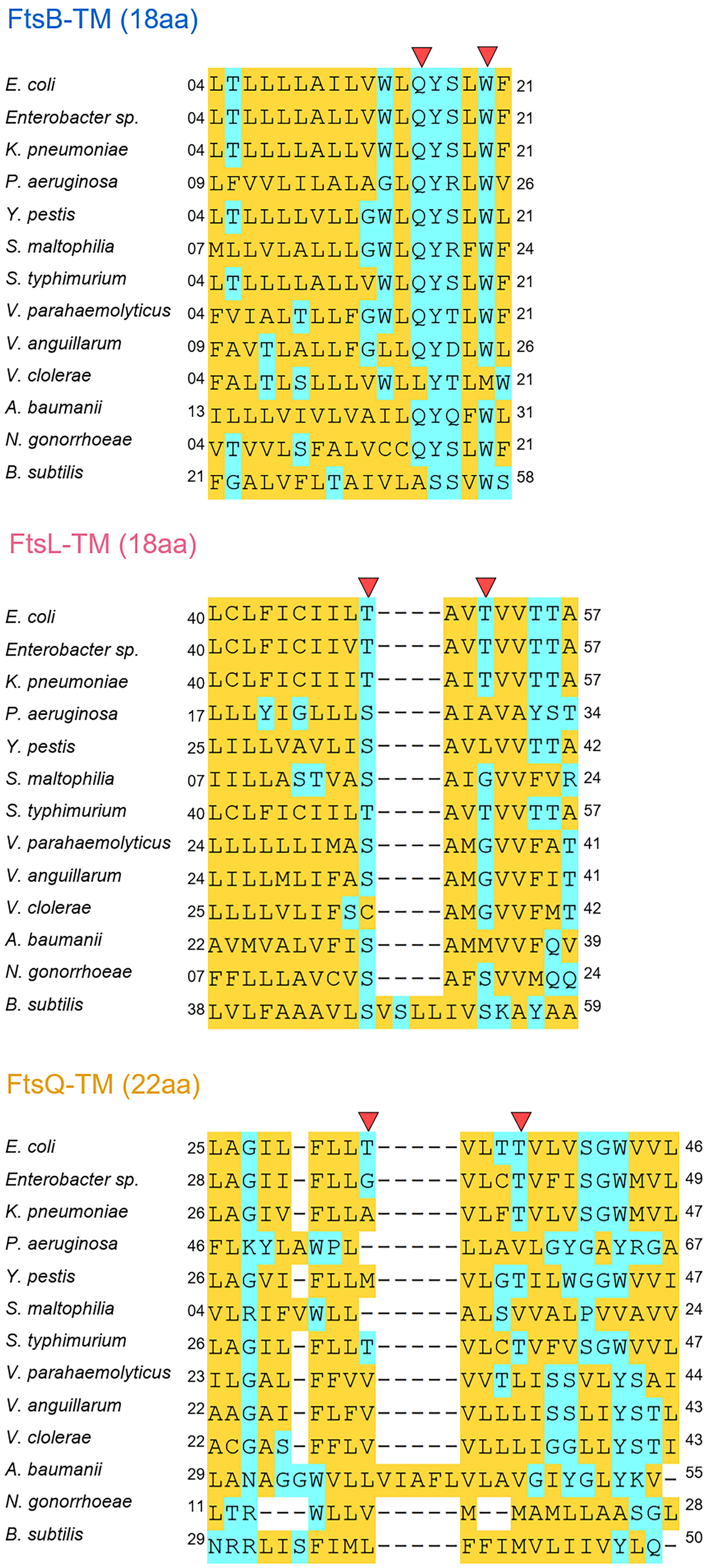

FtsB-TM (18aa)
E. coli 04 LTLLLLAILVWLQYSLWF 21
Enterobacter sp. 04 LTLLLLALLVWLQYSLWF 21
K. pneumoniae 04 LTLLLLALLVWLQYSLWF 21
P. aeruginosa 09 LFVVLILALAGLQYRLWV 26
Y. pestis 04 LTLLLLVLLGWLQYSLWL 21
S. maltophilia 07 MLLVLALLLGWLQYRFWF 24
S. typhimurium 04 LTLLLLALLVWLQYSLWF 21
V. parahaemolyticus 04 FVIALTLLFGWLQYTLWF 21
V. anguillarum 09 FAVTLALLFGLLQYDLWL 26
V. colerae 04 FALTLSLLLVWLLYTLMW 21
A. baumanii 13 ILLLVIVLVAILQYQFWL 31
N. gonorrhoeae 04 VTVVLSFALVCCQYSLWF 21
B. subtilis 21 FGALVFLTAIVLASSVWS 58
FtsL-TM (18aa)
E. coli 40 LCLFICIILT----AVTVVTTA 57
Enterobacter sp. 40 LCLFICIIVT----AVTVVTTA 57
K. pneumoniae 40 LCLFICIIIT----AITVVTTA 57
P. aeruginosa 17 LLLYIGLLLS----AIAVAYST 34
Y. pestis 25 LILLVAVLIS----AVLVVTTA 42
S. maltophilia 07 IILLASTVAS----AIGVVFVR 24
S. typhimurium 40 LCLFICIILT----AVTVVTTA 57
V. parahaemolyticus 24 LLLLLLIMAS----AMGVVFAT 41
V. anguillarum 24 LILLMLIFAS----AMGVVFIT 41
V. colerae 25 LLLLVLIFSC----AMGVVFMT 42
A. baumanii 22 AVMVALVFIS----AMMVVFQV 39
N. gonorrhoeae 07 FFLLLAVCVS----AFSVVMQQ 24
B. subtilis 38 LVLFAAAVLSVSLLIVSKAYAA 59
FtsQ-TM (22aa)
E. coli 25 LAGIL-FLLT-----VLTTVLVSGWVVL 46
Enterobacter sp. 28 LAGII-FLLG-----VLCTVFISGWMVL 49
K. pneumoniae 26 LAGIV-FLLA-----VLFTVLVSGWMVL 47
P. aeruginosa 46 FLKYLAWPL------LLAVLGYGAYRGA 67
Y. pestis 26 LAGVI-FLLM-----VLGTILWGGWVVI 47
S. maltophilia 04 VLRIFVWLL------ALSVVALPVVAVV 24
S. typhimurium 26 LAGIL-FLLT-----VLCTVFVSGWVVL 47
V. parahaemolyticus 23 ILGAL-FFVV-----VVTLISSVLYSAI 44
V. anguillarum 22 AAGAI-FLFV-----VLLLISSLIYSTL 43
V. colerae 22 ACGAS-FFLV-----VLLLIGGLLYSTI 43
A. baumanii 29 LANAGGWVLLVIAFLVLAVGIYGLYKV- 55
N. gonorrhoeae 11 LTR---WLLV-----M--MAMLLAASGL 28
B. subtilis 29 NRRLISFIML-----FFIMVLIIVYLQ- 50

Supplementary Fig. 5: Sequence conservation of the transmembrane (TM) region of FtsB, FtsL and FtsQ.

Protein sequences are aligned for the TM region of FtsB (top), FtsL (middle) and FtsQ (bottom) from various bacterial species as indicated. All residues are colored based on their hydrophobicity. Yellow: hydrophobic, Cyan: polar. Red triangles highlight key residues in the potential transmembrane interface as in Fig. 2b. The number of residues in each TM domain is also labeled with the protein.

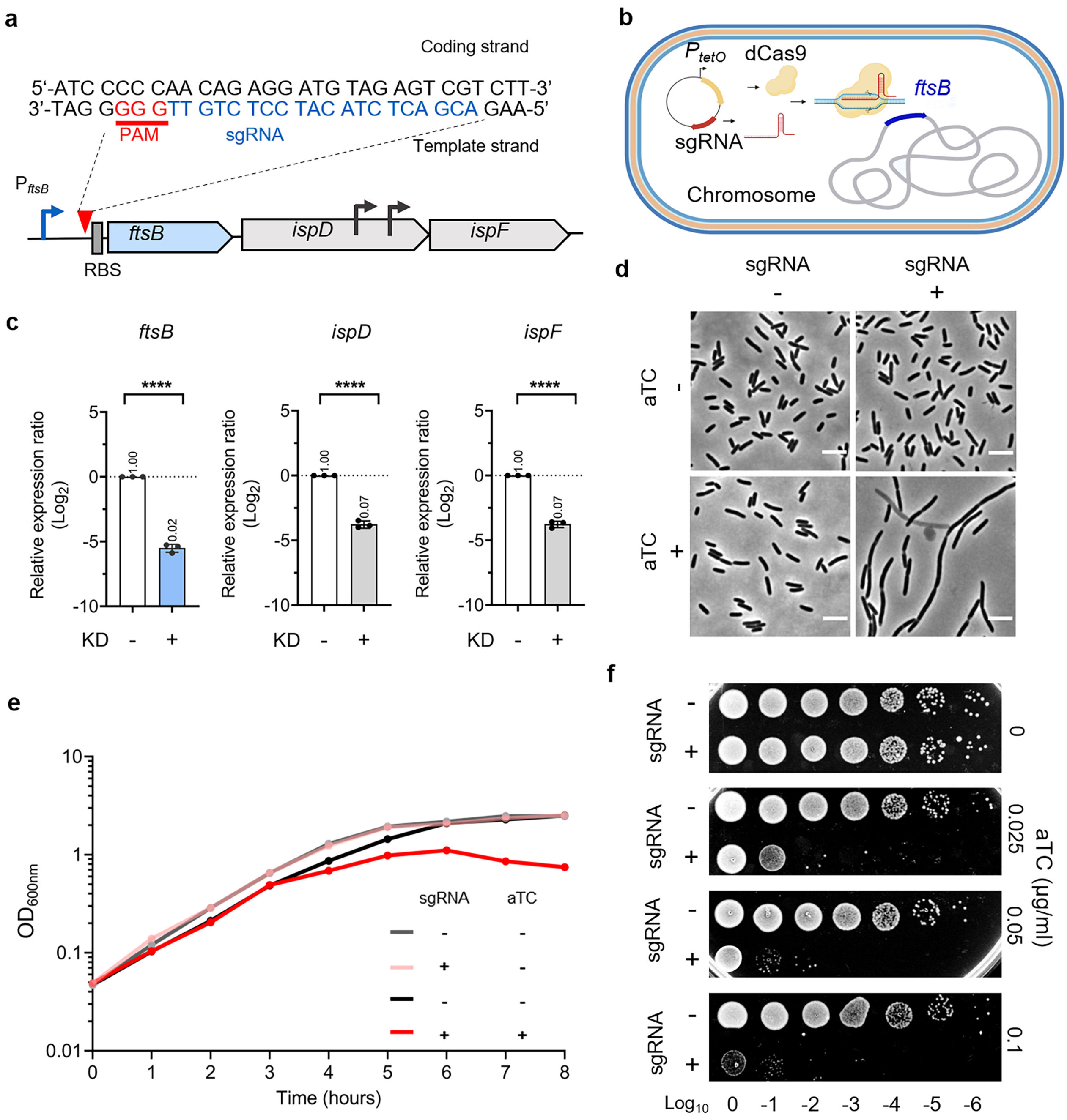

Supplementary Fig. 6: Knock-down (KD) of *ftsB* by CRISPRi.

a, Illustration of the sequence and the priming site of sgRNA for knocking down *ftsB*. **b**, A schematic view of the *ftsB*-KD experiments by the dCas9-sgRNA system. **c**, Relative expression of *ftsB*, *ispD* and *ispF* in the knock-down cell. Results are shown as the mean value with standard deviation (SD) from three biological replicates. Comparison of the paired samples was performed by two-tailed *t*-test. *P* <0.0001 (*ftsB*, *ispD*, *ispF*). **d**, Morphological changes of the *ftsB*-KD cells (right bottom) in comparison with the control cells. Scale bar 5 µm. **e**, Growth curves of the *E. coli* MC1000 strain carrying the empty vector as control and the sgRNA*ftsB* plasmid under non-induction and induction (0.05 µg/mL aTc) conditions. Curves are colored as indicated. The

experiment was performed with three biological replicates. **f**, The representative spot assay indicated the bacterial survival after *ftsB* depletion and induction of dCas9 expression at different concentrations of aTc. The dilution factors are labelled below each panel. The experiment was done with three independent replicates that showed similar results.

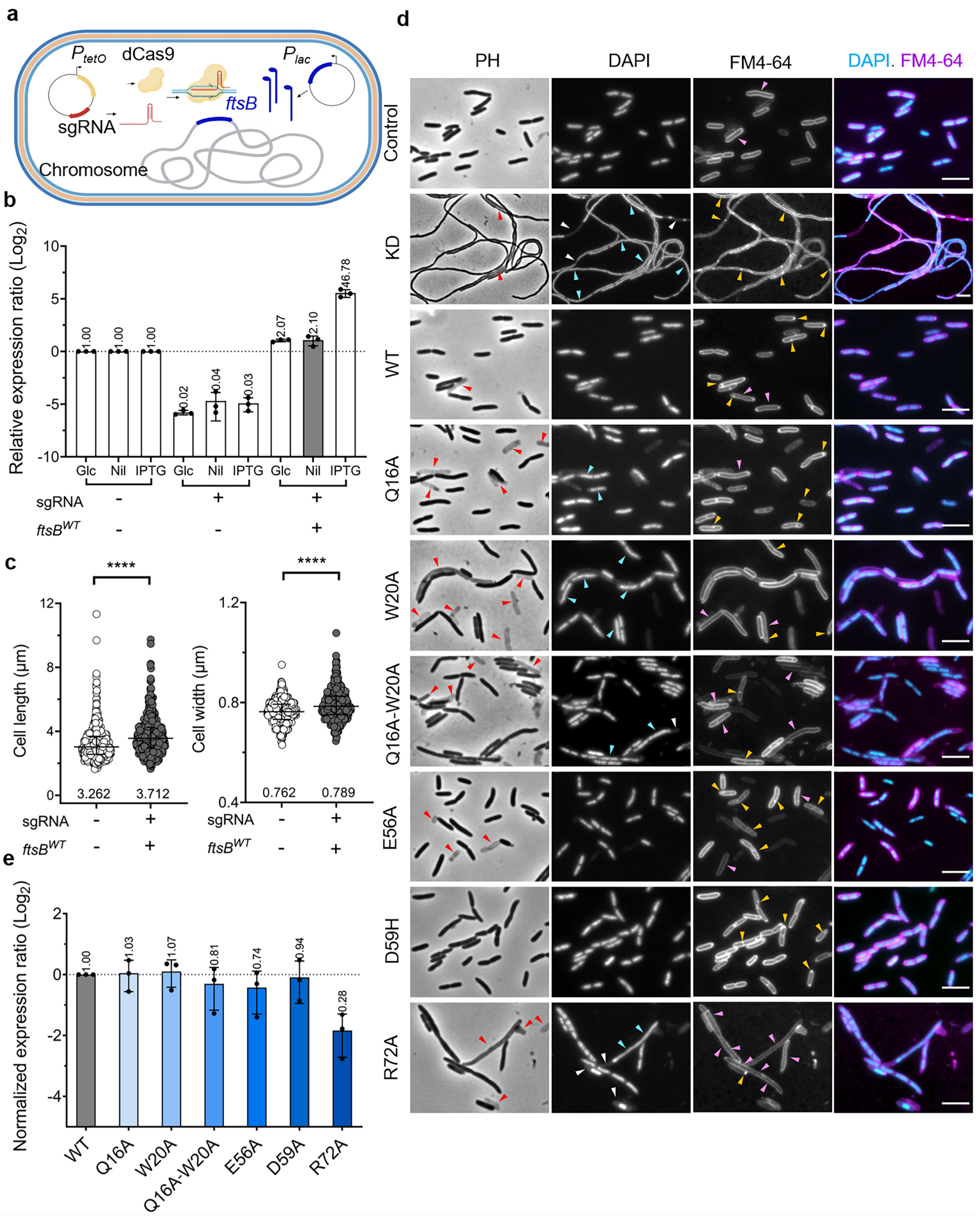

Supplementary Fig. 7: Complementation of the *ftsB* knock-down (KD) strain.

a, An illustration demonstrates complementation of the *ftsB*-KD strain using an *ftsB*-carrying plasmid. **b,** Relative expression of the *ftsB* mRNA levels in the control cells (no KD and no complement, two empty vectors), the *ftsB*-KD cells and the *ftsB*^{WT}-complemented cells under

different conditions (0.4% Glc, Nil (no repressor, no inducer) or 15 µM IPTG). The Nil condition of *ftsB*^{WT}-complemented cells (grey bar) was chosen for the subsequent experiments. The data are normalized against the control cell. Results are the mean value with SD of three biological replicates. The mean value is shown. **c,** Comparison of the length (left) and width (right) between cells with endogenous *ftsB* (white) and with the complementing *ftsB*^{WT} from the plasmid in the *ftsB*-knock-down strain (grey). Comparison was performed by two-tailed *t*-test. Data are presented as median with interquartile range. The median value is shown on bottom. Sample size n = 569 (white) and 564 (grey). $P < 0.0001$ (length, width). **d,** Morphology and fluorescent staining of the control, the KD, and the complemented cells by *ftsB*^{WT} (WT) and all mutants in this study. Arrows indicate the unsegregated chromosome (cyan), void of chromosome (white), lack of septum (magenta), and membrane punctate (orange) and the lysed cell (red). PH: phase contrast; Blue: DAPI for staining the genome; Purple: FM4-64 for staining the membrane. Scale bar, 5 µm. **e,** The *ftsB* expression levels in the complemented cells by the mutants as in (**d**). The expression ratios were normalized against the ratio measured in the complemented cells with the basal-level expression of *ftsB*^{WT} (grey bar) as in (**b**). For unknown reason, the expression level of *ftsB*^{R72A} was always low even with IPTG induction. Results are the mean value with SD of three biological replicates. Bars colored in blue gradients represent different mutants. Comparison was performed by One-way ANOVA test.

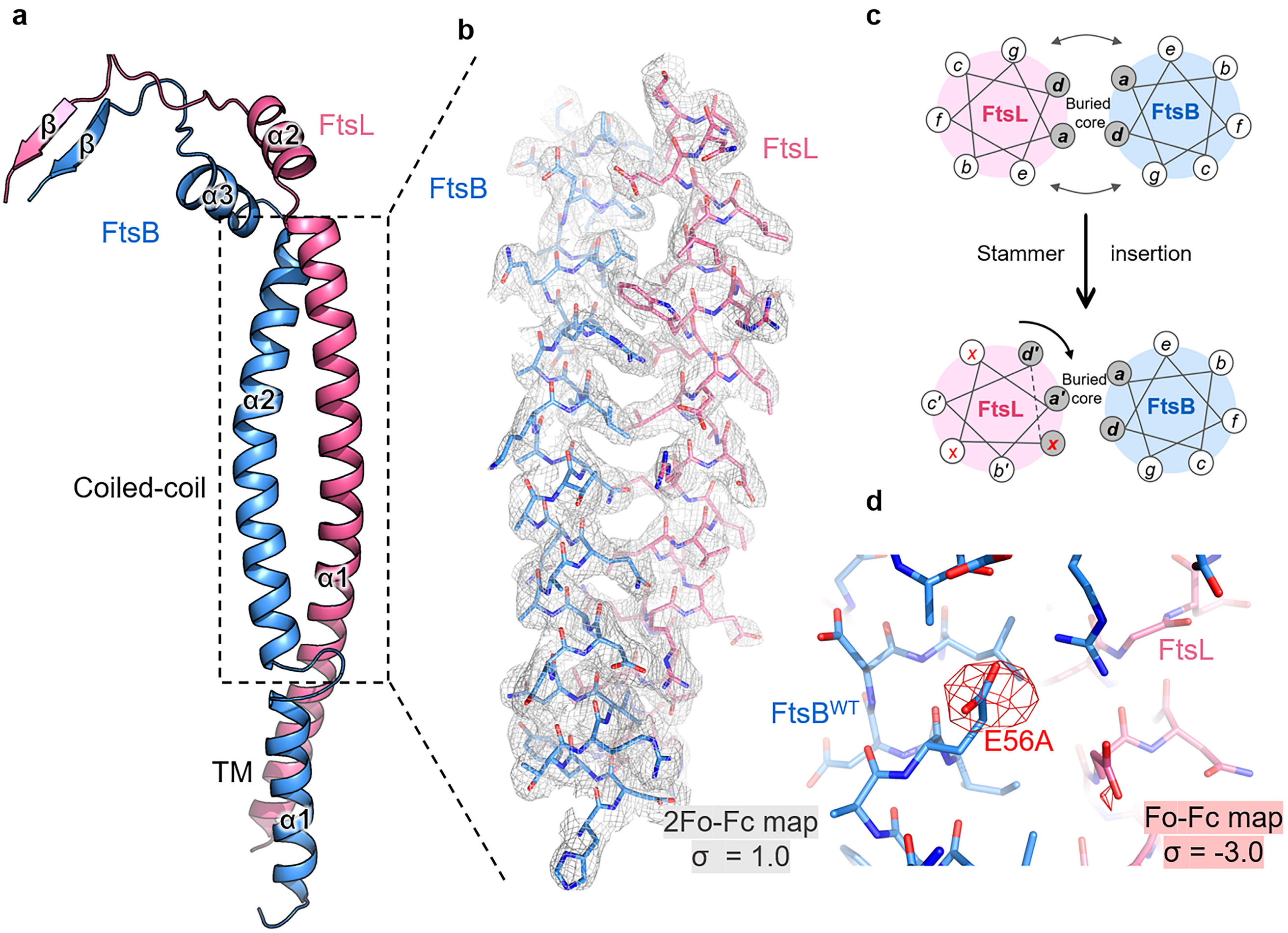

Supplementary Fig. 8: The detailed structure of the FtsBL coiled coil.

a, Overall structure of the FtsBL subcomplex, highlighting the heterodimer coiled coil with a box. **b**, The 2Fo-Fc electron density map (grey mesh) of the heterodimeric FtsBL coiled coil from the box in (**a**). FtsB and FtsL colored in blue and pink, respectively. **c**, The heptad *abcdefg* residues of FtsB (blue) and FtsL (pink) and the stammer insertion (red "x") of FtsL are illustrated in the helical-wheel projection. The core *a* and *d* positions are highlighted in dark grey. **d**, The Fo-Fc map (red, contoured at σ -3.0) around residue FtsB^{E56} of the FtsB^{E56A}LQ mutant complex when using the wild-type complex as the ensemble for molecular replacement.

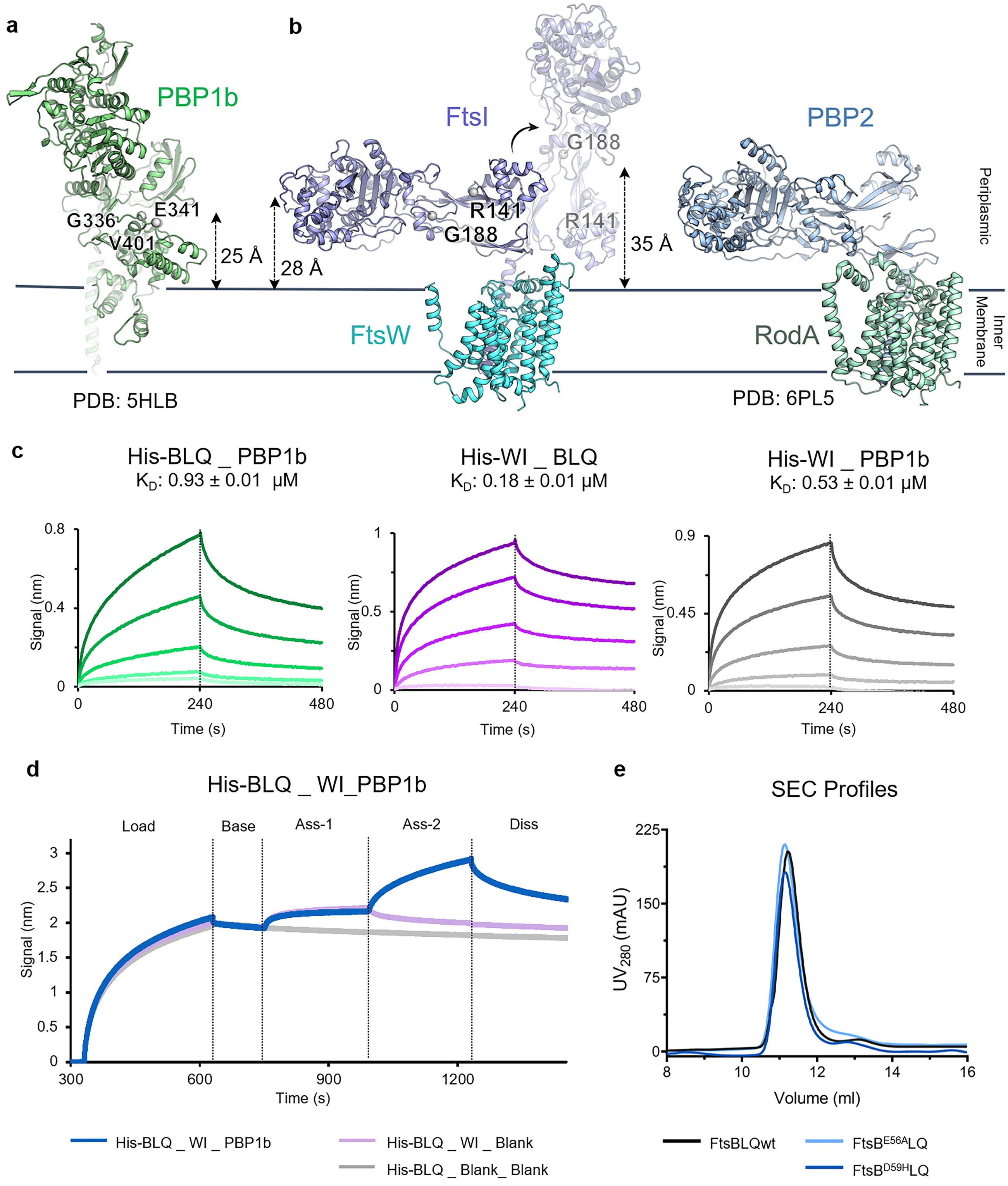

Supplementary Fig. 9: Structural and biochemical relationship between FtsBLQ and PG synthases.

a-b, Key residues of PBP1b (**a**, green) and FtsWI (**b**-left, cyan and purple) that potentially interact with FtsBLQ are used for distance measurements in **Fig. 4d**. Structures are shown in ribbons with PDB: 5HLB for PBP1b (**a**), and 6PL5 (**b**-right) for the PBP2-RodA complex, which is used for

modelling FtsW and FtsI (**b**-left) by Phyre2[1] with 100% confidence. Involved residues are drawn as grey Cα spheres and labeled. The membrane is shown as solid lines. The largest possible distance of the key residues in FtsWI was measured by flipping the periplasmic domain of FstI for 90° upwards. **c**, The kinetic binding curves of mutual interactions between FtsBLQ, PBP1b and FtsWI, with the dissociation constants (K_D) shown above. His-tagged proteins loaded on the biosensor were labeled accordingly. Analytes were serially diluted in the concentration range 0.016 µM - 4 µM. **d**, The biolayer interferometry curves of the sequential binding of FtsWI (Ass-1) and PBP1b (Ass-2) to the immobilized His-FtsBLQ (blue), with the controls shown in grey and light purple curves. **e**, SEC profiles of the FtsBLQ wild-type complex (black), the $FtsB^{E56A}$ (light blue) and the $FtsB^{D59H}$ (dark blue) mutant complexes.

Supplementary Table 1: X-ray crystallographic data collection and structural refinement statistics.

	FtsBLQ*	$FtsB^{D59H}LQ$	$FtsB^{E56A}LQ$
**Data collection**			
Space group	*I* 1 2 1	*C* 1 2 1	*I* 1 2 1
Cell dimensions			
a, *b*, *c* (Å)	101.09, 53.85, 177.42	199.61, 53.94, 101.85	100.29, 54.32 179.26
α, β, γ (°)	90, 93.93, 90	90, 116.67, 90	90, 94.51, 90
Resolution (Å)	88.50 - 3.10 (3.32 - 3.10) **	49.85– 2.90 (2.98-2.90)	90.33 - 3.30 (3.56 - 3.30)
R_{sym} or R_{merge}	0.162 (1.150)	0.126	0.07429 (0.6163)
I / σI	6.9 (1.6)	9.3	3.2 (1.2)
Completeness (%)	99.34 (99.77)	99.54 (99.79)	99.83 (100.00)
Redundancy	7.4	10.024	3.7
**Refinement**			
Resolution (Å)	34.45 - 3.10 (3.21 - 3.10)	49.85 - 3.04 (3.15 - 3.04	34.69 - 3.30 (3.42 - 3.30)
No. reflections	17613 (1742)	18951 (1868)	14814 (1476)
R_{work} / R_{free}	0.246/0.260	0.274 /0.302	0.278/0.304
No. atoms			
Protein	3281	3283	3277
Ligand/ion	0	0	0
Water	0	3	0
Ramachandran			
Favored (%)	98.76	94.29	96.53
Allowed (%)	1.24	5.71	3.23
Outliers (%)	0.00	0.00	0.25
Rotamer outliers (%)	0.00	0.00	0.28
B-factors			
Protein	109.0	137.0	127.0
Water		95.0	
R.m.s. deviations			
Bond lengths (Å)	0.002	0.002	0.004
Bond angles (°)	0.49	0.47	0.93

*2, 4, 1 xtals were used for FtsBLQ, $FtsB^{D59H}LQ$, $FtsB^{E56A}LQ$, respectively,

**Values in parentheses are for the highest-resolution shell.

Supplementary Table 2: Interacting residues in the FtsBLQ interface.

	FtsL	FtsB	FtsQ
**Interaction Site I and Site II**	V119		E225
	V119, V118, L117	V88	
	I117	L87	S250
	I117		V229, L226
	I117, N116, E115, Q114	R86	S250
	E115	Y85	G251, A252, Q233
	E115, Q114	F84	Y248
	E115	T83	V254
		E82	V254, A253, Y243
	L105	P80	
	M107	T78	
	H109, Q108, Q106	M77	
	H109	S76	
	H94	E74	
		N73	Y248
		R72	Y248, D245
	E88 (in coiled-coil)	R70	
		E69	R213, R196
	K104	E68	R247
	I100, V98	L67	
		E65	R196, Q200
**Coiled-coil**	L91	L60	
		D59	R196
		E56	R196, R213
	L84, W81	L53	
		Q52	R213
	E80	N50	
	W81	R49	
	L77	L46	
	E73	N43	
	L70, V71	Q39	
	L63	V32	
	T60	Y29	
	T56	G25	
**TM**	V53	L19	
	I46	L15	
	I46	L12	
	L42, P39	L8	
	P39	T5	

Supplementary Table 3: List of oligonucleotides used in this study.

Underlined: sequence of restriction site. Boxed: RBS sequence.

Oligonucleotides	Sequence (5'-3')	Purpose
ftsB-ftsL	<u>GAATTC</u>AATGGGTAAACTAACGCTGCTGTTGCTGGCTATTCTGGTCTGGCTACAGTATTCGCTGTGGTTCGGTAAGAACGGTATACATGACTATACCCGCGTCAATGATGATGTGGCGGCACAGCAAGCTACAAACGCGAAACTTAAAGCGCGAAACGATCAACTTTTTGCCGAAATTGACGATCTCAATGGCGGCCAGGAGGCGCTCGAAGAGCGTGCGCGTAATGAACTCAGCATGACCAGGCCGGGCGAAACTTTTTATCGTCTGGTGCCTGACGCGTCGAAGCGCGCACAGTCTGCGGGGCAAAACAATCGATAA<u>GTCGAC</u>AATAATTTTGTTTAACTTTAAGAAGGAGATATACATATGATTAATAAATTAACAGAAGCTCTAAGCAAAGTTAAAGGATCGATGGGAAGCCACGAGCGCCATGCATTGCCTGGTGTTATCGGTGACGATCTTTTGCGATTTGGGAAGCTGCCACTCTGCCTGTTCATTTGCATTATTTTGACGGCGGTGACTGTGGTAACCACGGCGCACCATACCCGTTTACTGACCGCTCAGCGCGAACAACTGGTGCTGGAGCGAGATGCTTTAGACATTGAATGGCGCAACCTGATCCTTGAAGAGAATGCGCTCGGCGACCATAGCCGGGTGGAAAGGATCGCCACGGAAAAGCTGCAAATGCAGCATGTTGATCCGTCACAAGAAAATATCGTAGTGCAAAAATAA<u>AAGCTT</u>	pRSF-FtsBL
**sgRNA*ftsB***	GGGTTGTCTCCTACATCTCAGCA	CRISPRi
**Q-F**	CGTC<u>AAGCTT</u>AATAATTTTGTTTAACTTTAAGAAGGAGATATACATATGTCGCAGGCTGCTCTG	pRSF-FtsBLQ
**Q-R**	GATC<u>GCGGCCGC</u>TCGCCTTGATCATTGTTGTTCTG	
**W-F**	GAAC<u>GGATCC</u>TATGCGTTTATCTCTCCCTC	pET-FtsW
**W-R**	GAGC<u>GAATTC</u>TCGCTTTCCTTGACCACTC	
**I-F**	CTTA<u>GACGTC</u>ATGAAAGCAGCGGCGAAAAC	pET-FtsWI
**I-R**	GAAT<u>CTCGAG</u>TTACGATCTGCCACCTGTC	
**BLQ +Thrombin_F**	CATCACCATCATCACCACAGCAGCGGCCTGGTGCCGCGCGGCAGCTCAATGGGTAAACTAACGCTG	pRSF-BLQ+ Thrombin
**BLQ +Thrombin_R**	CAGCGTTAGTTTACCCATTGAGCTGCCGCGCGGCACCAGGCCGCTGCTGTGGTGATGATGGTGATG	
**sgRNA*ftsB*-F**	<u>TAGT</u>ACGACTCTACATCCTCTGTT	pSOT 357
**sgRNA*ftsB*-R**	<u>AAAC</u>AACAGAGGATGTAGAGTCGT	
***Sal*I-P*ftsB*-F**	GCAT<u>GTCGAC</u>CGTAACTAATAATGAGATTATGTTC	

**_ftsB_-_BamH_I-R**	AGCTGGATCCCTGATTTATCGATTGTTTTGCC	pSOT 355/356/358
**_ftsB_-Q16A-R**	ACCGAACCACAGCGAATAGGCTAGCCAGACCAGAATAGC	pSOT 358
**_ftsB_-Q16A-F**	GCTATTCTGGTCTGGCTAGCCTATTCGCTGTGGTTCGGT	
**_ftsB_-W20A-R**	ACCGTTCTTACCGAAAGCCAGCGAATACTGTAGCCAGAC	pSOT 355
**_ftsB_-W20A-F**	TGGCTACAGTATTCGCTGGCTTTCGGTAAGAACGGTATACATG	
**_ftsB_-Q16AW20A-R**	ACCGAAAGCCAGCGAATAGGCTAGCCAGACCAGAATAGCCAG	pSOT 356
**_ftsB_-Q16AW20A-F**	GGCTAGCCTATTCGCTGGCTTTCGGTAAGAACGGTATACATGA	
**_EcoR_I-RBS-_ftsB_-F**	ACAGAATTCAGGGGGCAGGATGGGTAAACTAACGC	pSOT 390/360/362/ 363/364/388
**_ftsB_-_Hind_III-R**	CTGAAGCTTTTATCGATTGTTTTGCCCCGC	
**_EcoR_I-RBS-_ftsB_-R72A-F1**	CTATGACCATGATTACGAATTCAGGGGGCAGGATGGGTAAAC	pSOT 414
**_ftsB_-R72A-R1**	GCTGAGTTCATTAGCCGCACGCTCTTCGAGCGC	
**_ftsB_-R72A-F2**	GCTAATGAACTCAGCATGAC	pSOT 414
**_ftsB_-R72A-HindIII-R2**	GGCCAGTGCCAAGCTTTTATCGATTGTTTTGCCC	
**_cysG_-F**	TTAACCAGATCCCTGCTGCGG	RT-qPCR
**_cysG_-R**	ACCCGAATAGGCAGAGCAAC	
**_ftsB_-F**	CAGGATGGGTAAACTAACGC	RT-qPCR
**_ftsB_-R**	GTTTGTAGCTTGCTGTGCC	
**_ispF-F_**	CATTGGTGGCGTACGCATTC	RT-qPCR
**_ispF-R_**	GGGAACAGCTTGCCGATATC	
**_ispD-F_**	CATGGCAACCACTCATTTGG	RT-qPCR
**_ispD-R_**	GGCAATGACGACACGTTTCAC	

Supplementary Table 4. List of bacterial strains and plasmids used in this study.

Bacterial Strains/ Plasmids	Genotype	Source
E. coli **strain K-12 genome**	*F+ lambda+*	ATCC 10798D-5
**OverExpress™ C 43 (DE3)**	*F – ompT hsdSB (rB- mB-) gal dcm (DE3)*	Sigma
E. coli **strain MC1000**	*araD139 Δ(araABC-leu)7679 galU galK Δ(lac)X74 rpsL thi*	[2]
**pFD152**	P_{tet}-*dCas9* P_{pflB} *(BsaI) with RBS-4 oriT in pLZ12*	[3]
**pMLB1113**	*ColE1/pBR/pUC, bla*	[4]
**pKD3**	*oriR6Kγ, bla, rgnB, cat, FRT, bla*	[5]
**pRSFDuet**	RSF1030, *bla*	Novagen
**pRSF-FtsBL**	pRSFDuet $P_{T7/lac}$-*histag-ftsB*, $P_{T7/lac}$-*ftsL*	This study
**pRSF-FtsBLQ**	pRSFDuet $P_{T7/lac}$-*histag-ftsB*, $P_{T7/lac}$-*ftsL*, $P_{T7/lac}$-*ftsQ*	This study
**pRSF-FtsB^{D59H}LQ**	pRSFDuet $P_{T7/lac}$-*histag-ftsB*^{D59H}, $P_{T7/lac}$-*ftsL*, $P_{T7/lac}$-*ftsQ*	This study
**pRSF-FtsB^{E56A}LQ**	pRSFDuet $P_{T7/lac}$-*histag-ftsB*^{E56A}, $P_{T7/lac}$-*ftsL*, $P_{T7/lac}$-*ftsQ*	This study
**pRSF-BLQ +Thrombin**	pRSFDuet $P_{T7/lac}$-*histag-thrombin-ftsB*, $P_{T7/lac}$-*ftsL*, $P_{T7/lac}$-*ftsQ*	This study
**pETDuet**	ColE1, *bla*	Novagen
**pET-FtsWI**	pETDuet $P_{T7/lac}$-*histag-ftsW*, $P_{T7/lac}$-*ftsI*	This study
**pET15b-PBP1b**	pET15b $P_{T7/lac}$-*histag-thrombin-ponB*	[6]
**pSOT357**	pFD152, P_{tet}-*dCas9* P_{pflB}-*sgRNAftsB*	This study
**pSOT359**	pMLB1113, P_{lac}-*ftsB*	This study
**pSOT362**	pMLB1113, P_{lac}-*ftsB*^{Q16A}	This study
**pSOT363**	pMLB1113, P_{lac}-*ftsB*^{W20A}	This study
**pSOT364**	pMLB1113, P_{lac}-*ftsB*$^{Q16A\text{-}W20A}$	This study
**pSOT388**	pMLB1113, P_{lac}-*ftsB*^{E56A}	This study
**pSOT360**	pMLB1113, P_{lac}-*ftsB*^{D59H}	This study
**pSOT414**	pMLB1113, P_{lac}-*ftsB*^{R72A}	This study
**pSOT358**	pKD3, P_{ftsB}-*ftsB*^{Q16A}	This study

**pSOT355**	pKD3, P_{ftsB}-*ftsB*^{W20A}	This study
**pSOT356**	pKD3, P_{ftsB}-*ftsB*$^{Q16\text{-}W20A}$	This study

Supplementary References

1 Kelley, L. A., Mezulis, S., Yates, C. M., Wass, M. N. & Sternberg, M. J. E. The Phyre2 web portal for protein modeling, prediction and analysis. *Nature Protocols* **10**, 845-858, doi:10.1038/nprot.2015.053 (2015).

2 Casadaban, M. J. & Cohen, S. N. Analysis of gene control signals by DNA fusion and cloning in *Escherichia coli*. *J Mol Biol* **138**, 179-207, doi:10.1016/0022-2836(80)90283-1 (1980).

3 Depardieu, F. & Bikard, D. Gene silencing with CRISPRi in bacteria and optimization of dCas9 expression levels. *Methods* **172**, 61-75, doi:10.1016/j.ymeth.2019.07.024 (2020).

4 de Boer, P. A., Crossley, R. E. & Rothfield, L. I. A division inhibitor and a topological specificity factor coded for by the minicell locus determine proper placement of the division septum in *E. coli*. *Cell* **56**, 641-649, doi:10.1016/0092-8674(89)90586-2 (1989). Datsenko,

5 K. A. & Wanner, B. L. One-step inactivation of chromosomal genes in *Escherichia coli* K-12 using PCR products. *Proc Natl Acad Sci U S A* **97**, 6640-6645, doi:10.1073/pnas.120163297 (2000).

6 Sung, M.-T. *et al.* Crystal structure of the membrane-bound bifunctional transglycosylase PBP1b from *Escherichia coli*. *Proceedings of the National Academy of Sciences* **106**, 8824-8829, doi:10.1073/pnas.0904030106 (2009).

Uncropped data

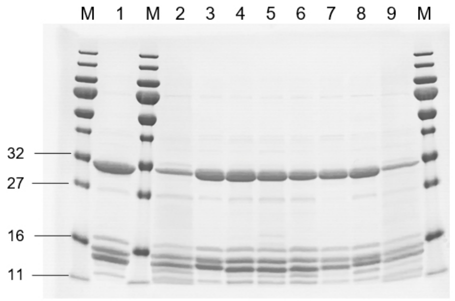

1: FtsBLQ-wild type
2: FtsBLQ^{Y248W}
3: FtsBLQ^{R196A}
4: FtsBLQ$^{E141V.S166R}$
5: FtsBLQ$^{K59E.R75E.Q76A}$
6: FtsBL^{I117P}Q
7: FtsB^{R86P}LQ
8: FtsB^{E82A}LQ
9: FtsB^{R72A}LQ

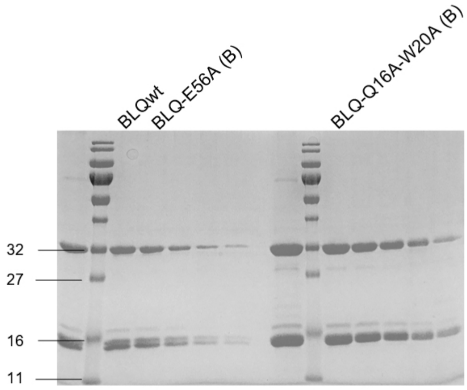

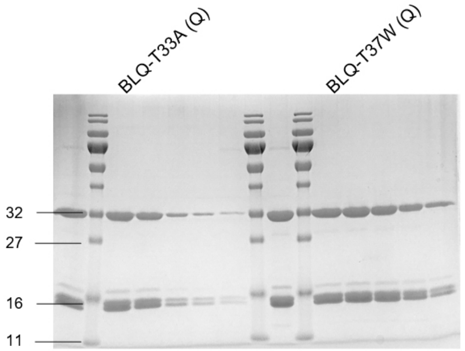

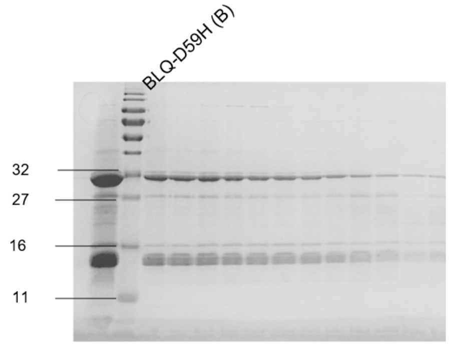

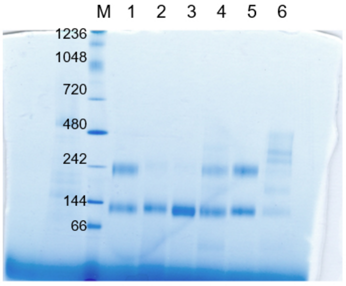

1: FtsBLQ wild type
2: FtsB^{E56A}LQ
3: FtsB^{D59H}LQ
4: FtsB$^{Q16A-W20A}$LQ
5: FtsBLQ^{T33A}
6: FtsBLQ^{T37W}

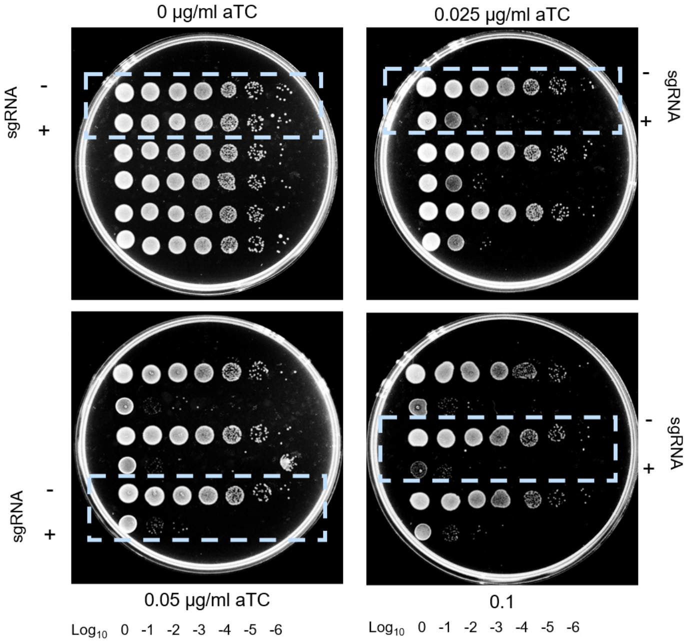

Supplement: Supplementary file 1 — Supplementary Information [file 41467_2023_37543_MOESM1_ESM.pdf]
